# Differential Antibody Responses to Conserved HIV-1 Neutralizing Epitopes in the Context of Multivalent Scaffolds and Native-Like gp140 Trimers

**DOI:** 10.1128/mBio.00036-17

**Published:** 2017-02-28

**Authors:** Charles D. Morris, Parisa Azadnia, Natalia de Val, Nemil Vora, Andrew Honda, Erick Giang, Karen Saye-Francisco, Yushao Cheng, Xiaohe Lin, Colin J. Mann, Jeffrey Tang, Devin Sok, Dennis R. Burton, Mansun Law, Andrew B. Ward, Linling He, Jiang Zhu

**Affiliations:** a Department of Immunology and Microbial Science, The Scripps Research Institute, La Jolla, California, USA; b Department of Integrative Structural and Computational Biology, The Scripps Research Institute, La Jolla, California, USA; c International AIDS Vaccine Initiative, Neutralizing Antibody Center and the Collaboration for AIDS Vaccine Discovery, The Scripps Research Institute, La Jolla, California, USA; d Center for HIV/AIDS Vaccine Immunology and Immunogen Discovery, The Scripps Research Institute, La Jolla, California, USA; e Ragon Institute of Massachusetts General Hospital, Massachusetts Institute of Technology and Harvard, Cambridge, Massachusetts, USA; George Washington University; Johns Hopkins Bloomberg School of Public Health

## Abstract

Broadly neutralizing antibodies (bNAbs) have provided valuable insights into the humoral immune response to HIV-1. While rationally designed epitope scaffolds and well-folded gp140 trimers have been proposed as vaccine antigens, a comparative understanding of their antibody responses has not yet been established. In this study, we probed antibody responses to the N332 supersite and the membrane-proximal external region (MPER) in the context of heterologous protein scaffolds and native-like gp140 trimers. Ferritin nanoparticles and fragment crystallizable (Fc) regions were utilized as multivalent carriers to display scaffold antigens with grafted N332 and MPER epitopes, respectively. Trimeric scaffolds were also identified to stabilize the MPER-containing BG505 gp140.681 trimer in a native-like conformation. Following structural and antigenic evaluation, a subset of scaffold and trimer antigens was selected for immunization in BALB/c mice. Serum binding revealed distinct patterns of antibody responses to these two bNAb targets presented in different structural contexts. For example, the N332 nanoparticles elicited glycan epitope-specific antibody responses that could also recognize the native trimer, while a scaffolded BG505 gp140.681 trimer generated a stronger and more rapid antibody response to the trimer apex than its parent gp140.664 trimer. Furthermore, next-generation sequencing (NGS) of mouse splenic B cells revealed expansion of antibody lineages with long heavy-chain complementarity-determining region 3 (HCDR3) loops upon activation by MPER scaffolds, in contrast to the steady repertoires primed by N332 nanoparticles and a soluble gp140.664 trimer. These findings will facilitate the future development of a coherent vaccination strategy that combines both epitope-focused and trimer-based approaches.

## IMPORTANCE

Both epitope-focused and trimer-based strategies are currently being explored in HIV-1 vaccine development, which aims to elicit broadly neutralizing antibodies (bNAbs) targeting conserved epitopes on the viral envelope (Env). However, little is known about the differences in antibody response to these bNAb targets presented by foreign scaffolds and native Env. In this study, a systematic effort was undertaken to design multivalent epitope scaffolds and soluble gp140.681 trimers with a complete antigenic surface, and to comparatively analyze the antibody responses elicited by these antigens to the N332 supersite and MPER in a mouse model. This study will inform both epitope-focused and trimer-based vaccine design and will facilitate integration of the two vaccine strategies.

## INTRODUCTION

Development of a broadly protective vaccine remains the highest priority in the global campaign against the AIDS epidemic. To achieve this goal, a rational vaccine strategy has been proposed that places a premium on broadly neutralizing antibodies (bNAbs) recognizing conserved epitopes on the envelope (Env) glycoprotein (1–4). A panel of diverse bNAbs has been identified from HIV-1-infected individuals (5–7), revealing multiple sites of viral vulnerability such as the CD4-binding site (CD4bs) and multiple glycan epitopes on gp120, the membrane-proximal external region (MPER) of gp41, and the quaternary gp120-gp41 interface (8). However, the unprecedented sequence diversity and structural metastability of Env have posed formidable obstacles to rational HIV-1 vaccine design (9, 10). The presence of immunodominant but nonneutralizing epitopes, in addition to poor recognition of bNAb epitopes due to glycan masking and restricted angles of approach, has also impeded vaccine development (11, 12).

Both epitope-focused and trimer-based strategies are currently being explored for HIV-1 vaccine design. For epitope-focused design, computational tools are often employed to graft an epitope of interest onto a heterologous protein scaffold (13, 14). Early epitope-scaffold designs focused on partial MPER epitopes targeted by NAbs 4E10, 2F5, and Z13 (15–19), in addition to the CD4 binding loop and outer domain exit loop recognized by NAb b12 (20). Recent success with respiratory syncytial virus (RSV) further confirmed that scaffolding is a promising solution to epitope-focused vaccine design (21). As more bNAbs are discovered (4), it is imperative to extend HIV-1 vaccine design efforts to those epitopes recognized by the “best-in-class” bNAbs. In particular, the N332 supersite at the base of variable loop 3 (V3) and MPER within the gp41 ectodomain (gp41_ECTO_) present prominent vaccine targets, as they are both recognized by bNAbs with extraordinary potency and breadth (22, 23). Since their primary antibody-binding sites are encoded within a linear peptide, these two epitopes can be readily grafted onto foreign scaffolds, although peripheral elements may also contribute to their bNAb recognition (24–26). Recently, Zhou et al. reported epitope scaffolds designed for three bNAb targets, including variable regions 1 and 2 (V1V2), the N332 supersite, and MPER (27). However, the lack of *in vivo* evaluation has hindered further development of these epitope scaffolds as immunogens.

In contrast to the reductionist approach of epitope scaffolding, trimer-based vaccine design aims to present bNAb epitopes in the native form of Env. To achieve this goal, uncleaved gp140 GCN4/foldon trimers and cleaved SOSIP trimers have been proposed (28–31). Notably, a soluble, cleaved BG505 SOSIP.664 trimer has demonstrated excellent structural and antigenic properties that mimic those of the native Env (32–37). The SOSIP design has been extended to multiple HIV-1 strains (38, 39), has allowed the incorporation of additional stabilizing mutations (40, 41), and has enabled the development of trimer immunogens targeting bNAb germline precursors (42). Other stabilized but cleavage-independent trimer designs, such as sc-gp140 (43) and NFL (44, 45), have also been reported. More recently, Kong et al. identified the N terminus of heptad repeat region 1 (HR1) as a major site of metastability and proposed an alternative strategy for trimer stabilization (46). Furthermore, the multivalent display of native-like gp140 trimers has been demonstrated for various nanoparticle platforms (47–49). Despite these advances, it remains unclear how the structural context of a bNAb epitope, presented by either heterologous scaffolds or native-like trimers, affects its antibody response. The lack of this knowledge has hindered the rational comparison of these two vaccine strategies and their potential for integration.

Here, we addressed this critical issue for the N332 supersite and MPER through immunogen design, structural and antigenic evaluation, mouse immunization, serum binding, and antibody repertoire analysis. We first utilized a scaffolding meta-server (50) to design a total of 21 new epitope scaffolds, which were either displayed on ferritin nanoparticle (for N332) or fused to the fragment crystallizable (Fc) region (for MPER). We also selected 5 trimeric scaffolds (TS) to present the BG505 gp140.681 trimer with a redesigned HR1 (46), resulting in stabilized gp140 trimers with a complete antigenic surface. All designs were assessed antigenically, while the N332 nanoparticles and scaffolded gp140 trimers were also validated structurally by electron microscopy (EM). A subset of antigens was then selected for immunization. For each epitope, we immunized three groups of BALB/c mice with either an epitope-focused antigen or a gp140 trimer and included a fourth group to test a trimer-prime/epitope-boost strategy. Serum binding against a panel of diverse antigens was evaluated to dissect the antibody responses, while deep sequencing provided further insights into the mouse splenic B-cell repertoires primed by the tested immunogens. Taken together, our key findings demonstrated that (i) one N332 nanoparticle elicited a consistent glycan-specific response cross-reactive with the same epitope on the native trimer; (ii) a scaffolded gp140.681 trimer, but not its parent gp140.664 trimer, induced a visible response against the trimer apex; and (iii) the MPER scaffolds showed robust B-cell activation, expanding antibody lineages with long heavy-chain complementarity-determining region 3 (HCDR3) loops. Overall, the structural context appears to exert a differential effect on the antibody response to a specific bNAb epitope, which is also influenced by the intrinsic features of the epitope and the immunization regimen.

## RESULTS

### Scaffolding the N332 supersite.

Structures of the N332 glycan-dependent bNAbs in complex with individual glycans, gp120 domains, and native-like gp140 trimers have provided important insights into how these bNAbs recognize a cluster of carbohydrates on gp120 and neutralize diverse HIV-1 isolates (23, 24, 51–54). The crystal structure of bNAb PGT128 bound to an engineered gp120 outer domain with a truncated V3 loop (eODmV3) established the *N*-linked glycans at positions N332 and N301, as well as the C-terminal V3 stem, as the primary antibody-binding sites (23). For other N332-dependent bNAbs, glycans in variable regions 1 and 2 (V1V2) may also be involved in epitope recognition (24, 25). The stems of the truncated V3 loop (residues 293 to 298 and 329 to 334) form a β-sheet stabilized by a disulfide bond (C296-C331), which harbors the glycans at N295, N301, and N332. This β-sheet can be used as a template to search for protein scaffolds that accommodate the PGT128 epitope.

**FIG 1:**
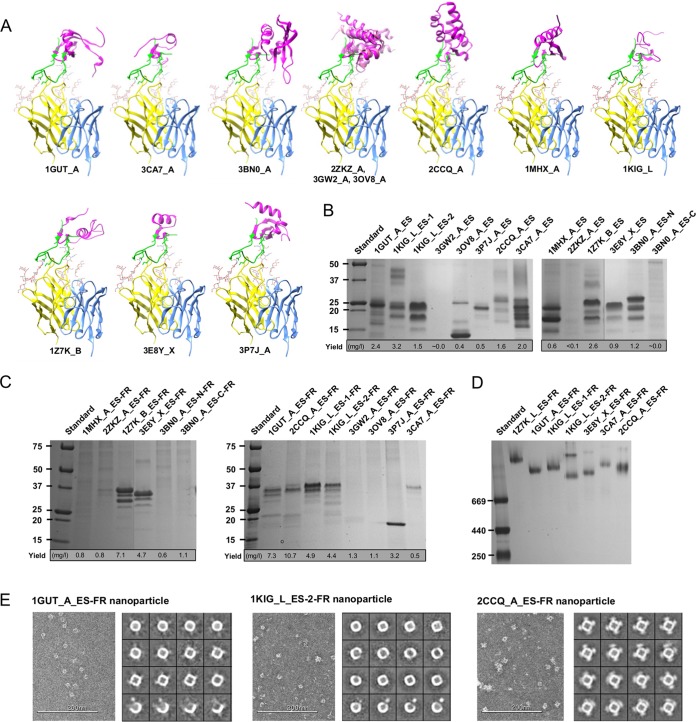
Expression of monomeric and particulate epitope scaffolds for the N332 supersite. (A) Twelve protein scaffolds identified by the scaffolding meta-server are superimposed onto the stems of a truncated V3 loop in complex with the broadly neutralizing antibody PGT128. All protein structures are shown as ribbon models, with the scaffold colored in magenta, epitope in green, PGT128 heavy chain in yellow, and light chain in cyan. The N301 and N332 glycans are shown as ball-and-stick models. Two scaffolds identified in the previous study (27), 1GUT_A and 3CA7_A, are included for comparison. The three structural homologs (2ZKZ_A, 3GW2_A, and 3OV8_A) are overlaid to facilitate structural comparison. (B) SDS-PAGE of 14 N332 scaffolds containing the truncated V3 loop under reducing conditions, with the estimated yield value indicated below the gel. (C) SDS-PAGE of 14 nanoparticles presenting the scaffolded N332 supersite under reducing conditions, with the estimated yield value indicated below the gel. (D) BN-PAGE of 7 expressed N332 nanoparticles. (E) Example micrographs and 2D class averages derived from negative-stain EM for three selected N332 nanoparticles, with a more complete EM analysis shown in Fig. S3. Design variants for scaffolds 3BN0_A and 1KIG_L were also included in the analysis whose results are shown in panels B to D.

A meta-server has been developed to combine six diverse scaffolding algorithms that demonstrated improved performance in the scaffold search (14, 50). This meta-server identified 643 scaffolds for the N332 supersite (see Materials and Methods). To evaluate the coverage of scaffold search, we calculated the number of scaffolds identified by each algorithm and the pairwise overlap (see Fig. S1A in the supplemental material). Improved coverage was observed, with each algorithm contributing 12% to 57% of the scaffolds. We then ranked the scaffolds according to the number of algorithms (votes) by which they were identified (Fig. S1B). Eight of the 11 previously reported functional scaffolds (27) were found in the groups with 3 to 5 votes, suggesting the presence of high-quality scaffolds. From these three groups, we manually identified 10 new scaffolds with diverse folds ranging from 30 to 99 amino acids (aa) in size, all of which contained an exposed β-hairpin matching the N- and C-terminal V3 stems with a C_α_ root-mean-square deviation (RMSD) of 1.4 Å or less (Fig. 1A and S1C). The V3 loop (residues N295 to S334), excluding its crown motif, was then grafted onto these 10 scaffolds along with 1GUT_A and 3CA7_A from the previous study (27) (see Table S1 in the supplemental material). The resulting epitope scaffolds are indicated here by the suffix “_ES” (e.g., 1GUT_A_ES).

10.1128/mBio.00036-17.1 FIG S1  N332 epitope-scaffold design and experimental validation by Western blotting. (A) Coverage matrix of protein scaffolds identified by the scaffolding meta-server. The number of scaffolds identified by each of the six structural alignment algorithms is shown in blue, while the overlap of two algorithms is shown in italics. (B) Consensus analysis of protein scaffolds identified by the scaffolding meta-server. The number of scaffolds is plotted as a function of the number of algorithms (or votes) by which a scaffold is identified. The meta-server-identified scaffolds are shown in cyan, with 11 previously reported functional scaffolds (27) shown in light green and 10 manually selected scaffolds shown in orange. (C) Summary of 10 protein scaffolds identified by the scaffolding meta-server, with the two previously reported functional scaffolds (1GUT_A and 3CA7_A) included for comparison. All the parameters listed were derived from the scaffolding meta-server. (D) Immunoblot analysis of three selected N332 scaffolds (1GUT_A_ES, 1KIG_L_ES-2, and 2CCQ_A_ES). Results of reduced SDS-PAGE, Western blot detection with 6×His epitope mouse antibody (red), and Western blot detection with PGT128 human IgG (green) are shown in three panels from the left to the right. Of note, the faint bands observed for 1KIG_L_ES-2 suggest that this antigen is more sensitive to the conditions used in Western blot analysis than the other two antigens. Download FIG S1, TIF file, 0.9 MB. Copyright © 2017 Morris et al. 2017 Morris et al. This content is distributed under the terms of the Creative Commons Attribution 4.0 International license.

10.1128/mBio.00036-17.9 TABLE S1  Amino acid sequences of rationally designed HIV-1 antigens. Download TABLE S1, DOCX file, 0.1 MB. Copyright © 2017 Morris et al. 2017 Morris et al. This content is distributed under the terms of the Creative Commons Attribution 4.0 International license.

Following computational design, His-tagged epitope scaffolds were transiently expressed in HEK-293 F cells with kifunensine to produce a Man_8/9_ glycan at the N332 position (23). Of note, the kifunensine treatment would also affect other glycosylation sites on the grafted N332 supersite. Expressed antigens were purified using a nickel affinity column and characterized by sodium dodecyl sulfate polyacrylamide gel electrophoresis (SDS-PAGE). All epitope scaffolds with sufficient yield showed multiple bands corresponding to various glycoforms at N295, N301, and N332 (Fig. 1B), as further indicated by Western blotting of three antigens using an anti-His-tag antibody and PGT128 (Fig. S1D). Of note, a design variant was also generated for both 1KIG_L and 3BN0_A, showing improved purity and/or yield. We then investigated particulate display for all designed N332 scaffolds. Virus-like particles (VLPs), with a dense layer of surface antigens, can induce more potent immune responses than subunit vaccines (55–59). Small, self-assembling nanoparticles thus provide a versatile platform to display various vaccine antigens, allowing streamlined production and evaluation (48, 50). Since only 4 of 20 N332 epitope scaffolds could be displayed on a ferritin nanoparticle previously (27), a set of criteria in favor of particulate display (14) were applied to scaffold selection in this study. For all the newly designed epitope scaffolds, the C terminus of each antigen was fused to the N terminus of a ferritin subunit (Asp5) with a 5-aa flexible linker. The resulting constructs are indicated here by the suffix “-FR” (e.g., 1GUT_A_ES-FR). In cases where the fusion protein is capable of forming a nanoparticle, 24 copies of the epitope scaffold can be displayed on the surface (Fig. S2). All nanoparticle constructs were transiently transfected in HEK-293 F cells with kifunensine and purified using a *Galanthus nivalis* lectin (GNL) column. Under denaturing conditions, SDS-PAGE showed sufficient yield and the proper molecular weight for 7 of the 14 nanoparticle constructs (Fig. 1C). Blue native polyacrylamide gel electrophoresis (BN-PAGE) also demonstrated molecular weights consistent with well-formed nanoparticles (Fig. 1D), which were further visualized by negative-stain EM (Fig. 1E and S3). Of note, 2CCQ_A_ES forms discernible spikes on the nanoparticle surface, likely due to its large size and compact protein fold.

10.1128/mBio.00036-17.2 FIG S2  Surface and structure models of scaffold-ferritin nanoparticles. Each nanoparticle displays 24 copies of a scaffold, with the protein backbone shown as ribbon models within the molecular surface (both in gray) and the epitope transplantation site marked with red dots on each scaffold. For the three structural homologs (2ZKZ_A-FR, 3GW2_A-FR, and 3OV8_A-FR), the nanoparticles are overlaid with the epitope transplantation sites circled by dotted red lines. Download FIG S2, TIF file, 1.1 MB. Copyright © 2017 Morris et al. 2017 Morris et al. This content is distributed under the terms of the Creative Commons Attribution 4.0 International license.

10.1128/mBio.00036-17.3 FIG S3  Negative-stain EM analysis of N332 nanoparticles. Micrographs and 2D class averages derived from negative-stain EM for seven N332 nanoparticles, including (A) 1GUT_A_ES-FR, (B) 3CA7_A_ES-FR, (C) 1KIG_L_ES-1-FR, (D) 1KIG_L_ES-2-FR, (E) 2CCQ_A_ES-FR, (F) 3E8Y_X_ES-FR, and (G) 1Z7K_B_ES-FR, are shown. Download FIG S3, TIF file, 4.8 MB. Copyright © 2017 Morris et al. 2017 Morris et al. This content is distributed under the terms of the Creative Commons Attribution 4.0 International license.

**FIG 2:**
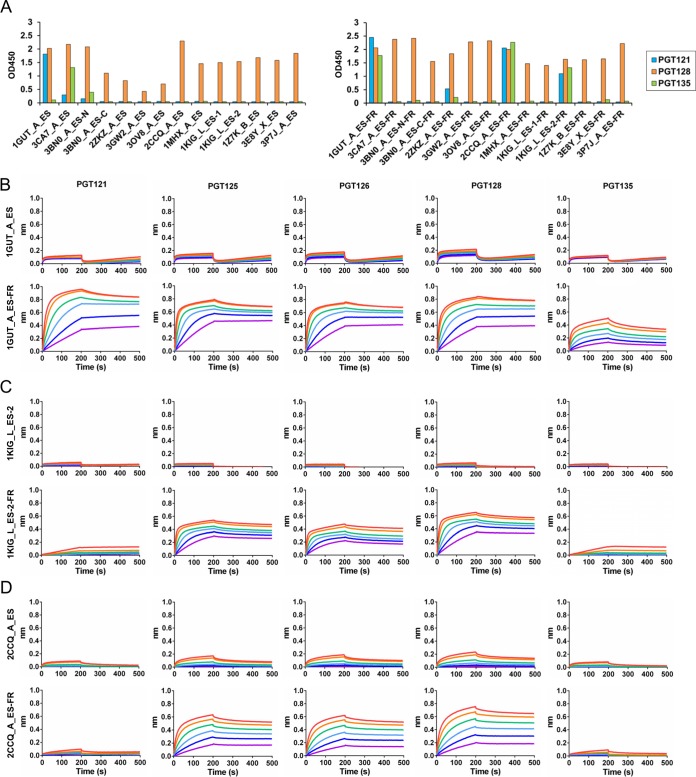
Antigenic profiles of N332 epitope scaffolds and nanoparticles. (A) Antigenic evaluation of 14 epitope scaffolds (left) and nanoparticles (right) presenting the N332 supersite against three representative N332-dependent bNAbs—PGT121, PGT128, and PGT135. The OD_450_ value at the highest antigen concentration (50 μg/ml) is shown, with the ELISA curves presented in Fig. S4. (B to D) Octet binding of monomeric N332 scaffolds and their respective nanoparticles to a panel of 5 N332-dependent bNAbs is shown for 1GUT_A_ES and 1GUT_A_ES-FR (B), 1KIG_L_ES-2 and 1KIG_L_ES-2-FR (C), and 2CCQ_A_ES and 2CCQ_A_ES-FR (D). Sensorgrams were obtained from an Octet RED96 instrument using a titration series of six starting at the maximum of 500 nM for monomeric epitope scaffolds and 50 nM for nanoparticles, respectively.

We next assessed the binding of monomeric and particulate scaffold antigens to N332-dependent bNAbs PGT121, PGT128, and PGT135 (60) by enzyme-linked immunosorbent assay (ELISA) (Fig. 2A and S4). For monomers, the two best designs selected from the previous study (27) could be recognized by 2 of 3 bNAbs, whereas all new designs bound only to PGT128, with the exception of 3BN0_A_ES-N, which also bound weakly to PGT135. In contrast, three N332 nanoparticles—1GUT_A_ES-FR, 2CCQ_A_ES-FR, and 1KIG_L_ES-2-FR—bound to all three bNAbs with comparable values for optical density at 450 nm (OD_450_). The lack of binding to PGT121 and PGT135 by most monomers suggests that recognition of the N332 supersite by these two bNAbs is highly dependent on the glycoform and environment, as revealed recently (24, 25, 51, 52). For the three nanoparticles with binding, it is plausible that some glycans from neighboring epitope scaffolds on the particle surface are recruited by PGT121 and PGT135 as “substitutes” to the required Env glycans, consistent with the promiscuous nature of glycan recognition by these bNAbs. Biolayer interferometry (BLI) was then used to characterize the binding kinetics of 3 monomeric antigens and their particulate counterparts for an array of PGT bNAbs (Fig. 2B to D). Overall, the binding results from BLI were consistent with those from ELISA but also revealed critical differences between these designs. In particular, 1GUT_A_ES exhibited the most pronounced binding to all three bNAbs when displayed on a nanoparticle, with a fast on-rate and slow dissociation curves similar to those previously reported (27). While 1KIG_L_ES-2-FR and 2CCQ_A_ES-FR also bound to PGT121 and PGT135, they showed lower affinities and different dissociation patterns. Of note, 2CCQ_A_ES displayed higher affinities than 1KIG_L_ES-2 for two other members of the PGT128 class (PGT125 and PGT126), in both monomeric and particulate forms (Fig. 2C and D).

10.1128/mBio.00036-17.4 FIG S4  Antigenic screening of N332-focused immunogens. Data represent results obtained from the ELISA analysis of 14 pairs of monomeric and particulate N332 scaffolds binding to 3 representative N332-dependent bNAbs (PGT121, PGT128, and PGT135). Binding curves for monomeric N332 scaffolds (ES) and ferritin-fusion (FR) nanoparticles are shown in blue and magenta, respectively. EC_50_ values are not labeled, as this ELISA was performed only for the initial screening of N332-focused antigens. The OD_450_ value at the highest antigen concentration (50 μg/ml) is plotted in Fig. 2. Download FIG S4, TIF file, 1.2 MB. Copyright © 2017 Morris et al. 2017 Morris et al. This content is distributed under the terms of the Creative Commons Attribution 4.0 International license.

In this study, systematic design and screening generated new N332-focused antigens that can be recognized by diverse N332-dependent bNAbs. It also appears that some critical features of the N332 supersite on native Env can be recapitulated by particulate display of the scaffolded V3 base, suggesting that glycan promiscuity may be utilized to design broadly reactive antigens targeting this site of viral vulnerability.

### Scaffolding the full-length MPER.

This conserved C-terminal segment of gp41_ECTO_ has long been considered an HIV-1 vaccine target (61). Various scaffold antigens have been designed for the overlapping MPER epitopes recognized by NAbs 2F5, 4E10, and Z13 (15–19). However, these early-generation MPER-directed NAbs suffered from autoreactivity and limited potency (62). The identification of 10E8, a bNAb with an exceptional neutralization breadth of ~98% (22), has renewed hope for development of an MPER-focused HIV-1 vaccine. However, lipid binding (26) and reactivity with a host protein (63) were recently noted for 10E8. In the crystal structure (22), each asymmetric unit contains two copies of the MPER:10E8 complex with a Cα RMSD of 1.6 Å between the two MPER conformations in chain E (residues 656 to 684) and chain F (residues 659 to 684). The angles between the N- and C-terminal helices in the two MPER conformations differ by 45°, suggesting some degree of structural plasticity.

**FIG 3:**
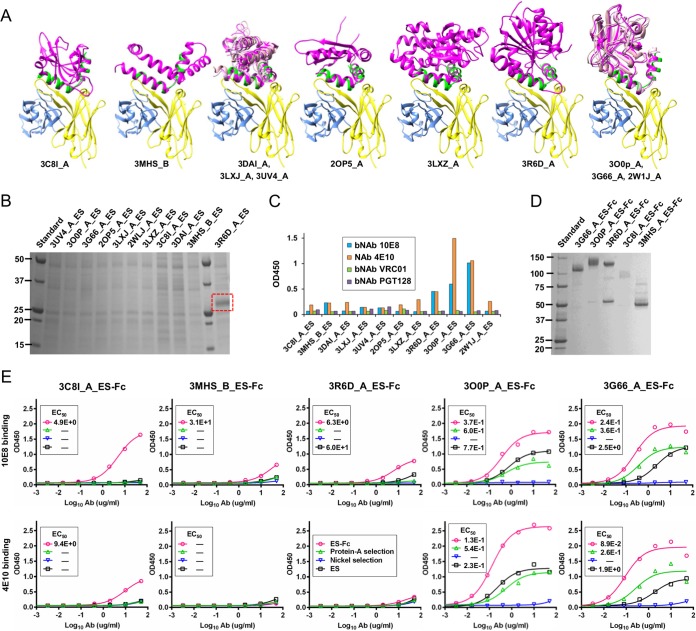
Expression of monomeric and Fc-fused MPER epitope scaffolds. (A) Eleven protein scaffolds identified by the scaffolding meta-server are superimposed onto the MPER epitope in complex with the broadly neutralizing antibody 10E8. All protein structures are shown as a ribbon model, with the scaffold colored in magenta, epitope in green, 10E8 heavy chain in yellow, and light chain in cyan. Two scaffolds identified in the previous study (27), 3C8I_A and 3MHS_B, are included for comparison. The two groups of structural homologs are overlaid to facilitate structural comparison. (B) SDS-PAGE of 11 nonredundant MPER scaffolds containing the full-length epitope under reducing conditions, with the visible 3R6D_A_ES band outlined in a red, dashed-line box. (C) Antigenic evaluation of 11 MPER scaffolds against bNAb 10E8 and NAb 4E10, with CD4bs-directed bNAb VRC01 and N332-dependent bNAb PGT128 included as controls. The OD_450_ value at the specified antigen concentration (10 μg/ml) is shown. (D) SDS-PAGE of 5 Fc-fused MPER scaffolds under nonreducing conditions. (E) ELISA binding of monomeric MPER scaffolds, bivalent Fc-fused MPER scaffolds, and samples collected during Fc cleavage to bNAb 10E8 and NAb 4E10 for scaffolds 3C8I_A, 3MHS_B, 3R6D_A, 3O0P_A, and 3G66_A (from left to right). The EC_50_ values are labeled for all ELISA plots in panel E except for instances in which the highest OD_450_ value was below 0.1 or in the cases of ambiguous data fitting.

A scaffolding meta-server identified 390 and 994 scaffolds matching the two MPER conformations present in the crystal structure (Fig. S5A and S5C). We assessed the scaffolding coverage, selecting 6 and 5 scaffolds to present the MPER conformation in chains E and F, respectively (Fig. 3A and S5B and S5D). Further analysis revealed two groups of structural homologs: 3DAI_A, 3LXJ_A, and 3UV4_A share sequence homology of 23% to 71% and a C_α_ RMSD of 0.5 to 2.3 Å (Fig. S5A), whereas 3O0P_A, 3G66_A, and 2W1J_A form a group with sequence homology of 51% to 63% and a C_α_ RMSD of 1.0 to 1.4 Å (Fig. S5C). Of note, the only two full-length MPER-matching scaffolds in the previous study (27), 3C8I_A and 3MHS_B, were also identified here by the meta-server. A total of 11 nonredundant scaffolds, including the 2 previously reported scaffolds, were selected for epitope grafting. The 10E8-interacting residues were then transplanted onto these 11 scaffolds with further mutations to minimize unfavorable interactions between the graft and the scaffold (Table S1).

10.1128/mBio.00036-17.5 FIG S5  MPER epitope-scaffold design. (A and C) Scaffold identification for the MPER conformations in chain E and chain F, respectively. (Left) Coverage matrix of protein scaffolds identified by the scaffolding meta-server. The number of scaffolds identified by each of the six structural alignment algorithms is shown in blue, while the overlap of two algorithms is shown in italics. (Middle) Consensus analysis of protein scaffolds identified by the scaffolding meta-server. The number of scaffolds is plotted as a function of the number of algorithms (or votes) by which a scaffold is identified. The meta-server-identified scaffolds are shown in cyan, with 2 previously reported functional scaffolds (27) shown in light green and manually selected scaffolds shown in orange (6 for chain E and 5 for chain F, respectively). (Right) Pairwise sequence and structure comparison within a group of structural homologs containing 3DAI_A, 3LXJ_A, and 3UV4_A for chain E and 3O0P_A, 3G66_A, and 3W1J_A for chain F, respectively. The number of residues, the Cα-RMSD, and sequence identity are listed. (B and D) Summary of protein scaffolds identified by the scaffolding meta-server for the MPER conformations in chains E and F, respectively. Two previously reported scaffolds (3C8I_A and 3MHS_B) are included for comparison. All the parameters listed were derived from the scaffolding meta-server. Download FIG S5, TIF file, 1 MB. Copyright © 2017 Morris et al. 2017 Morris et al. This content is distributed under the terms of the Creative Commons Attribution 4.0 International license.

Following computational design, His-tagged MPER scaffolds were transiently expressed in HEK-293 F cells and purified using a nickel affinity column. SDS-PAGE revealed low to undetectable expression for all constructs except for 3R6D_A_ES (Fig. 3B). However, the poor antigen yield was not surprising given that all 11 constructs expressed nearly full-length MPER with large, hydrophobic patches. The 10E8 bNAb and the 4E10 NAb were then utilized to probe the MPER epitope presented on various protein scaffolds by ELISA. Three designs—3R6D_A_ES, 3G66_A_ES, and 3O0P_A_ES—can be well recognized by 10E8 and 4E10, in contrast to their negligible binding to the CD4bs-directed bNAb VRC01 and the N332-dependent bNAb PGT128 (Fig. 3C). The remaining designs exhibited minimal binding to 10E8 and 4E10, highlighting the difficulty of scaffolding the full-length, hydrophobic MPER. We then investigated the utility of bivalent Fc to improve the expression and solubility of the 3 MPER scaffolds with notable binding for 10E8 and 4E10, along with the two previous designs (27). These constructs are indicated here by the suffix “-Fc” (e.g., 3R6D_A_ES-Fc). SDS-PAGE showed visible bands for all Fc-attached MPER scaffolds (Fig. 3D). The impact of Fc fusion on antigenicity was evaluated by ELISA, which showed improved 10E8 and 4E10 binding for the Fc-fused antigens in comparison to their monomeric forms (Fig. 3E). Overall, 3G66_A_ES-Fc and 3O0P_A_ES-Fc retained the highest affinity for both antibodies, consistent with the 50% effective concentration (EC_50_) values and the finding that these two scaffolds are structural homologs (Fig. S5C).

Our results confirm that the full-length MPER presented on rationally designed scaffolds can be readily recognized by bNAb 10E8 and NAb 4E10, which both require the C terminus of MPER to neutralize diverse HIV-1 isolates (22). We also demonstrated that the expression and antigenicity of MPER scaffolds can be significantly improved by Fc fusion, as shown for designs 3O0P_A_ES-Fc and 3G66_A_ES-Fc. The effect of viral lipids on the recognition of MPER by 10E8 and 4E10 (26) can be further explored for these antigens through liposome display (64).

### Scaffolding the BG505 gp140.681.R1 trimer.

**FIG 4:**
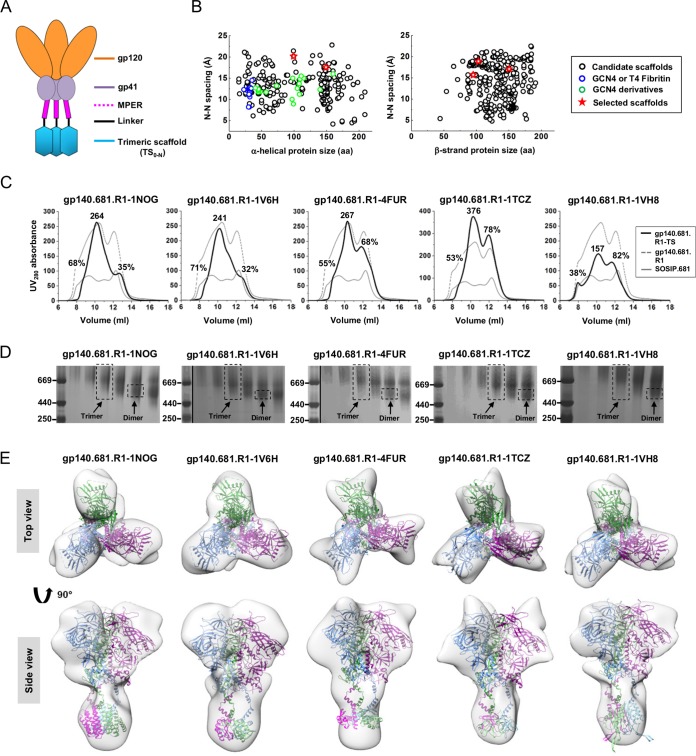
Design and characterization of scaffolded BG505 gp140.681 trimers. (A) Schematic design of scaffolded gp140.681 trimer, with gp120 in orange, gp41_ECTO_ (excluding MPER) in purple, MPER in magenta, flexible linker in black, and trimeric scaffold (TS) in cyan. (B) Distributions of identified TS domains with the N terminus being an α-helix (left) and a β-strand (right). The average spacing between two N termini within a TS domain is plotted against the protein size (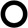), with GCN4 and T4 fibritin shown as blue circles, GCN4 derivatives as green circles, and 5 selected TS domains as red stars. (C) SEC profiles of 5 scaffolded BG505 gp140.681.R1 trimers (R1 stands for a redesigned HR1 bend in gp41_ECTO_) (46) from a Superdex 200 10/300 GL column. The absolute UV_280_ value is labeled for the trimer peak (at 10.5 ml), while the percentage relative to the UV value of the trimer peak is labeled for both aggregate (at 9 ml) and dimer/monomer (at 12 ml) peaks. The SEC profiles of unscaffolded gp140.681.R1 (gray dashed line) and SOSIP.681 trimers (gray solid line) are included for comparison. (D) BN-PAGE of 5 scaffolded gp140.681.R1 trimers following SEC. The trimer fraction used for EM analysis is circled by black dashed lines, with the expected positions of trimer and dimer bands labeled on the gel. (E) 3D reconstructions of scaffolded gp140.681.R1 trimers derived from negative-stain EM. The trimer densities are shown by a gray transparent surface, with the fitted structures of scaffolded trimers modeled upon the crystal structure of the BG505 SOSIP. 664 trimer (PDB 4TVP), with three chains shown in cyan, green, and magenta, respectively. Both the top and side views seen after fitting the structural model into the density are shown.

As demonstrated for the KNH1144 gp140 trimers, the inclusion of MPER increased the aggregation of produced Env proteins whereas its absence improved trimer solubility at the cost of reduced stability for the resulting SOSIP.664 gp140 trimer (65). In the early gp140 designs, trimerization motifs such as GCN4 and foldon were used to stabilize the uncleaved gp140 trimers with full-length gp41_ECTO_ in a soluble form (28–30), suggesting that MPER capping may prevent aggregation. Recently, Kong et al. reported an HR1-redesigned gp140.664 trimer with substantially improved purity and a more stable gp41 (46), termed gp140.664.R1 here. In the present study, we hypothesized that a trimeric scaffold (TS) can be used to stabilize an HR1-redesigned gp140.681 trimer to present all bNAb epitopes in their native-like, trimeric form (Fig. 4A). A database search identified 721 TS domains, 285, 322, and 19 of which contained an α-helix (H), a β-strand (E), and a loop (L) at the N terminus, respectively (Fig. 4B and S6A and B). We manually selected two TS domains from the H group and three from the E group, each with a size of 100 to 150 aa and an N-terminal spacing of 16 to 20 Å (Fig. S6C). Five fusion constructs (Table S1) were designed that contained a BG505 gp140.681 with a redesigned HR1 bend (46), a restriction site (AS), and a TS domain. These scaffolded full-length gp140 trimer constructs are termed gp140.681.R1-TS here.

10.1128/mBio.00036-17.6FIG S6 Structural design and negative-stain EM analysis of scaffolded BG505 gp140.681.R1 trimers. (A) Distribution of identified TS domains categorized on the basis of the N-terminal secondary structure element (SSE). The TS domains identified by database search are shown in cyan, with GCN4 or T4 fibritin proteins shown in light green and 5 manually selected TS domains shown in orange. (B) Distribution of TS domains, with the N-terminal SSE being characterized as a loop. The average spacing between two N termini within a TS domain is plotted against the protein size (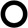), with GCN4 and T4 fibritin shown as blue circles, GCN4 derivatives as green circles, and selected TS domains as red stars. (C) Structures of 5 scaffolded gp140.681.R1 trimers modeled upon the crystal structures of BG505 SOSIP trimer (PDB ID: 4TVP), MPER in the 10E8-bound structure (PDB ID: 4G6F), and the respective TS domains. (D) Reference-free 2D class averages of scaffolded gp140.681.R1 trimers containing TS domains 1NOG, 1V6H, 4FUR, 1TCZ, and 1VH8. (E) The estimated resolutions of EM reconstructions were calculated from the Fourier shell correlation (FSC) using a cutoff of 0.5 for 5 scaffolded gp140.681.R1 trimers containing TS domains 1NOG (~20 Å), 1V6H (~21 Å), 4FUR (~22 Å), 1TCZ (~21 Å), and 1VH8 (~20 Å). Download FIG S6, TIF file, 3.2 MB.Copyright © 2017 Morris et al.2017Morris et al.This content is distributed under the terms of the Creative Commons Attribution 4.0 International license.

The gp140.681.R1-TS constructs were expressed transiently in HEK-293 F cells with cotransfected furin. The secreted Env proteins were purified using a GNL column followed by size exclusion chromatography (SEC) on a Superdex 200 10/300 column. One liter of expression was sufficient to generate 7 to 9 mg of total protein for further characterization. The gp140.681.R1-TS trimers were first compared to the unscaffolded gp140.681.R1 and SOSIP.681 trimers based on the UV absorbance values at 280 nm (UV_280_) obtained from SEC (Fig. 4C). As previously described, the UV value of the trimer peak was used as an indicator of the trimer yield, with the aggregate and dimer/monomer peaks measured as percentages of their UV values versus that of the trimer peak (46). Overall, the unscaffolded trimers displayed a large amount of aggregates as well as dimers and monomers, with gp140.681.R1 showing significantly improved yield and a notable trimer peak (Fig. 4C). Among the scaffolded trimers, the two H-group designs—gp140.681.R1-1NOG and gp140.681.R1-1V6H trimers—appeared to be the best performers, showing comparably low dimer/monomer peaks. In contrast, all three E-group designs displayed a 2-fold increase in the dimer/monomer peak but a notable reduction in aggregates. The trimer-containing fractions eluted at 10.25 to 10.75 ml showed a diffuse band in BN-PAGE for the five gp140.681.R1-TS constructs, consistent with the presence of aggregates (Fig. 4D). Negative-stain EM yielded three-dimensional (3D) reconstructions for all five gp140.681.R1-TS trimers (Fig. 4E and S6D and E). Of note, the 20-Å reconstruction of gp140.681.R1-1NOG exhibited a well-defined trimer apex and a distal TS domain, while the 21-Å reconstruction of gp140.681.R1-1V6H showed a similar structure but with less-defined features in the apex and gp41 (Fig. 4E).

**FIG 5:**
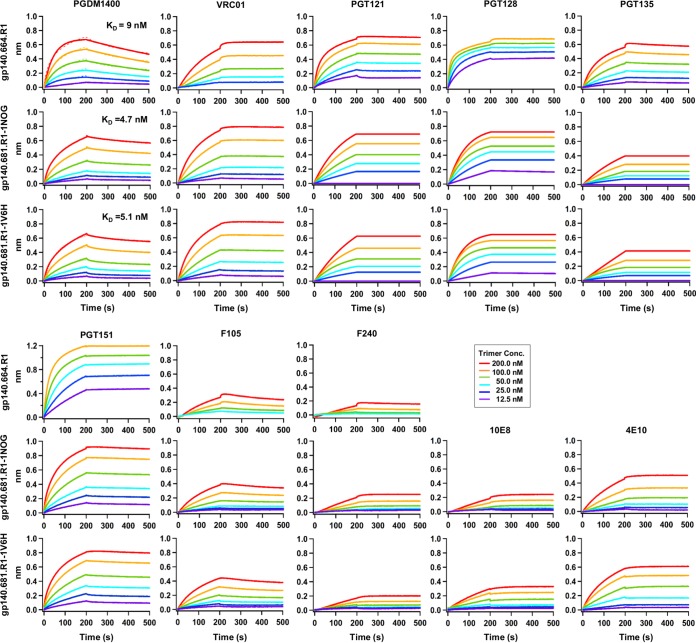
Antigenic profiles of scaffolded BG505 gp140.681.R1 trimers. Antibody binding kinetics were measured for two constructs using a panel of representative bNAbs and non-NAbs, including MPER-specific bNAb 10E8 and NAb 4E10. Sensorgrams were obtained from an Octet RED96 instrument using a trimer titration series of six concentrations (200 to 12.5 nM by 2-fold dilution). *K*_*D*_ values calculated from 1:1 global fitting are labeled for the V1V2 apex-directed bNAb PGDM1400. The construct gp140.664.R1 is an HR1-redesigned BG505 gp140 trimer (46) included here for comparison with two scaffolded gp140.681.R1 trimers.

We next investigated the effect of MPER and TS domain on trimer antigenicity by BLI using a panel of bNAbs and non-NAbs (Fig. 5). To this end, the two scaffolded gp140.681.R1 trimers from the H-group were compared to the parent gp140.664.R1 trimer (46). We first utilized bNAb PGDM1400 (66) to probe the trimeric V1V2 apex. Remarkably, both scaffolded trimers displayed nanomolar affinities for this bNAb (equilibrium dissociation constant [*K*_*D*_] = ~5 nM), indicating an intact trimer apex. For VRC01, which represents a class of CD4bs-directed bNAbs (67–70), the scaffolded trimers showed increased on-rates compared to the parent trimer (*k*_on_= ~3.0 × 10^4^ versus ~8.7 × 10^3^ 1/Ms). In contrast, both scaffolded trimers displayed reduced on-rates for the N332-dependent bNAbs PGT121, PGT128, and PGT135 (60). For PGT151, which targets the quaternary gp120-gp41 interface (71, 72), the scaffolded trimers showed binding profiles similar to those seen with the parent trimer, with subtle differences in their on-rates. We then measured trimer binding to two non-NAbs. Both scaffolded trimers bound to CD4bs-specific F105, as well as F240 (which targets an immunodominant epitope in cluster I of gp41_ECTO_), showing signals that were slightly higher than the parent trimer but with similar kinetics. Overall, the gp140.681.R1-1NOG and -1V6H trimers displayed native-like antigenic profiles largely resembling those of the gp140.664.R1 trimer (46). Lastly, we tested trimer binding for two MPER-specific antibodies. The two scaffolded trimers showed preferential binding to NAb 4E10, although they both bound to bNAb 10E8 and NAb 4E10 with slow on-rates and flat dissociation curves. This was not unexpected as 4E10 recognizes a shorter fragment of MPER, making it less constrained by the angle of approach (22). In addition, gp140.681.R1-1V6H displayed stronger binding to 10E8 and 4E10 than gp140.681.R1-1NOG, suggesting that a small TS domain such as 1V6H may permit greater MPER accessibility.

In comparison to the soluble, native-like gp140.664.R1 trimer, our results demonstrate that a gp140 trimer with a complete gp41_ECTO_ can be presented by rationally designed scaffolds without impairing its structure and antigenicity. Having an N-terminal helix in the TS domain appears to exert a more positive effect on the folding and purity of scaffolded gp140 trimers, consistent with the fact that MPER is anchored by a transmembrane helix in the context of a membrane-bound gp160 Env spike. Scaffolded gp140.681.R1 trimers thus provide promising immunogens that can present all bNAb targets in native-like conformations.

### Dissecting N332-specific antibody responses.

Recently, Hu et al. reported a detailed analysis of murine antibody responses to BG505 SOSIP.664 trimer (73). In this study, we utilized a mouse model with a simple regimen to investigate the N332 supersite-directed antibody responses in the context of scaffolds and gp140 trimers. Briefly, we immunized three groups of BALB/c mice with two N332 nanoparticles—1GUT_A_ES-FR and 1KIG_L_ES-2-FR—and the native-like gp140.664.R1 trimer (46), with a fourth group included to test a trimer-prime/epitope-boost strategy. In total, four groups of mice were immunized with antigens formulated in AddaVax adjuvant at weeks 0, 3, and 6, with blood and spleen harvested at week 8 for analysis. When the mouse antisera were tested against six tier 2 HIV-1 isolates (see Materials and Methods), no neutralization (indicated by a 50% inhibitory concentration [IC_50_] value of ≥50) was observed, consistent with the previous report (73). Based on this finding, we utilized serum binding to probe the early antibody response.

**FIG 6:**
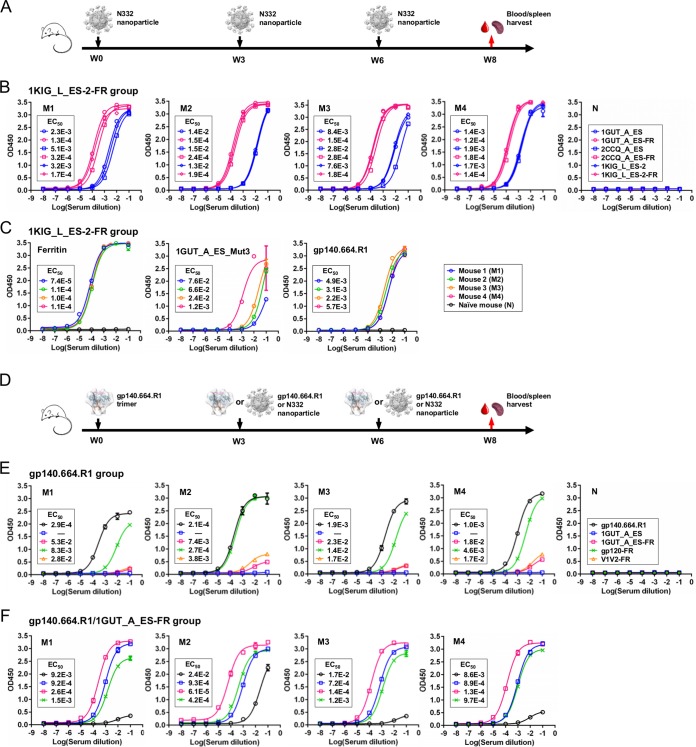
Analysis of the antibody response to the N332 supersite in mouse antisera. (A) Schematic view of the immunization regimen to test two N332 nanoparticles in BALB/c mice (W, week). (B) ELISA binding of mouse antisera from the 1KIG_L_ES-2-FR group to 3 pairs of monomeric and particulate N332 scaffolds (M, treated mouse; N, naïve mouse). (C) ELISA binding of mouse antisera from the 1KIG_L_ES-2-FR group to ferritin, a mutated 1GUT_A_ES antigen—1GUT_A_ES_Mut3—with three glycan knockout mutations (N295A, N301A, and N332A), and the native-like gp140.664.R1 trimer (46). (D) Schematic view of the immunization regimen to test the gp140.664.R1 trimer and a trimer-prime/epitope-boost strategy in BALB/c mice. (E) ELISA binding of mouse antisera to the gp140.664.R1 trimer, a pair of monomeric and particulate N332 scaffolds, a V1V2 nanoparticle, and a gp120 nanoparticle (48) for 4 subjects from the trimer-alone group. (F) ELISA binding of mouse antisera to the same set of antigens as described for panel E for 4 subjects from the heterologous prime/boost group. The immunogens and the regimen details are labeled on the diagram for panels A and D. A naïve mouse sample was included in the ELISA as a control, as shown in panels B and E. The EC_50_ values are labeled for all ELISA plots in panels B, C, E, and F, except for instances in which the highest OD_450_ value was below 0.1 or in the cases of ambiguous data fitting.

We first examined the antibody response to the grafted N332 supersite using antisera elicited by two N332 nanoparticles (Fig. 6A). Three N332 scaffolds—1GUT_A_ES, 1KIG_L_ES-2, and 2CCQ_A_ES—and their nanoparticles were utilized to probe the epitope specificity by ELISA (Fig. 6B and S7A). Surprisingly, serum binding to the N332 glycan epitope presented by three structurally distinct scaffolds showed negligible differences in EC_50_ values and binding profiles. Overall, antisera showed stronger binding to nanoparticles than to monomers, as indicated by a 10- to 100-fold difference in their EC_50_ values. This pattern suggests that a significant fraction of the elicited response may be directed to ferritin. To examine this possibility, we utilized ferritin as a coating antigen to test all four mouse samples from each nanoparticle group, using a naïve mouse sample as a control. Indeed, we observed nearly identical levels of strong ferritin binding for all but the naïve sample (Fig. 6B and C and S7C), which was expected since the two N332 scaffolds are relatively small and ferritin accounts for a majority of the nanoparticle surface. To probe the glycan-specific antibody response, we utilized a “negative” antigen (1GUT_A_ES_Mut3) with three alanine mutations at N295, N301, and N332, respectively (Fig. S7B). The antisera from both nanoparticle groups exhibited differential binding to this mutant antigen, indicating that the glycan-specific responses may vary between animals and that a fraction of the elicited antibodies may recognize only the peptide portion of this epitope (Fig. 6C and S7C). We then tested serum binding to the gp140.664.R1 trimer (46). All antisera from the 1KIG_L_ES-2-FR group bound to this native trimer at a scale similar to that seen with the epitope scaffolds (Fig. 6C), whereas the 1GUT_A_ES-FR group exhibited differential binding profiles (Fig. S7C). Taken together, both N332 nanoparticles elicited antibody responses that could recognize the N332 supersite on the native trimer but in a scaffold-specific manner. We also created two naked scaffolds without the grafted N332 supersite (1GUT_A and 1KIG_L) to analyze the scaffold-directed response in mouse antisera (Fig. S8A). The naked scaffolds were validated by ELISA, which showed minimal binding to bNAb PGT128. The mouse antisera displayed stronger binding to the epitope scaffolds than to the naked scaffolds, with 10- to 100-fold lower EC_50_ values, suggesting that the scaffold response represents only a fraction of the total antibody response elicited by each N332 nanoparticle.

10.1128/mBio.00036-17.7 FIG S7  Additional analysis of the antibody response to the N332 supersite in mouse antisera. (A) ELISA binding of mouse antisera from the 1GUT_A_ES-FR group to 3 pairs of monomeric and particulate N332 scaffolds. (B) ELISA binding of 1GUT_A_ES and 1GUT_A_ES_Mut3, which is a mutant antigen containing three glycan knockout mutations (N295A, N301A, and N332A), to 3 representative N332-dependent bNAbs (PGT121, PGT128, and PGT135). (C) ELISA binding of mouse antisera from the 1GUT_A_ES-FR group to ferritin, 1GUT_A_ES_Mut3, and the native-like gp140.664.R1 trimer (46). The EC_50_ values are labeled for all ELISA plots, except in instances in which the highest OD_450_ value was below 0.1 or in the cases of ambiguous data fitting. Download FIG S7, TIF file, 0.9 MB. Copyright © 2017 Morris et al. 2017 Morris et al. This content is distributed under the terms of the Creative Commons Attribution 4.0 International license.

10.1128/mBio.00036-17.8 FIG S8  Analysis of the scaffold-directed antibody response in mouse antisera. (A) ELISA binding of N332 scaffolds and their respective naked scaffolds to bNAb PGT128 (top panel), mouse antisera from the 1GUT_A_ES-FR group (middle panel), and mouse antisera from the 1KIG_L_ES-2-FR group (bottom panel). (B) ELISA binding of MPER scaffolds and their respective naked scaffolds to bNAb 10E8 (top panel), mouse antisera from the 3O0P_A_ES-Fc group (middle panel), and mouse antisera from the 3G66_A_ES-Fc group (bottom panel). The EC_50_ values are labeled for all ELISA plots, except in instances in which the highest OD_450_ value was below 0.1 or in the cases of ambiguous data fitting. Download FIG S8, TIF file, 1.6 MB. Copyright © 2017 Morris et al. 2017 Morris et al. This content is distributed under the terms of the Creative Commons Attribution 4.0 International license.

We next examined the N332-specific antibody responses in the trimer and trimer-prime/epitope-boost groups (Fig. 6D). Overall, the gp140.664.R1 trimer, despite outstanding structural and antigenic profiles (46), did not induce a strong antibody response in comparison to the N332 nanoparticles (Fig. 6E). We then utilized 1GUT_A_ES and 1GUT_A_ES-FR to probe the N332-specific antibody response in the antisera of the trimer group. Surprisingly, ELISA showed negligible signals for both antigens (Fig. 6E), in contrast to the notable trimer reactivity observed for the antisera elicited by two N332 nanoparticles (Fig. 6C and S7C). We also utilized two newly developed nanoparticles (48) as coating antigens in ELISA analysis. While the trimer-elicited antibody response appeared to recognize a BG505 gp120 nanoparticle with comparable EC_50_ values, we observed only a weak apex-directed response, as indicated by minimal binding to a nanoparticle presenting trimeric V1V2 (Fig. 6E). We speculate that the V3 tip and other immunodominant epitopes on the native trimer might have diverted antibody responses away from the bNAb epitopes (73). Of note, the naïve mouse sample exhibited no binding to any antigens. Lastly, antisera from the trimer-prime/epitope-boost group showed a rather strong antibody response to the boost antigen (N332 nanoparticle) but not to the prime antigen (native trimer), with the exception of one subject (Fig. 6F). All mice in this group showed serum binding to the gp120 nanoparticle at a scale similar to that seen with the monomeric 1GUT_A_ES.

In summary, while N332 nanoparticles elicit differential antibody responses to the N332 supersite presented on foreign protein scaffolds and a native trimer (46), the trimer alone cannot induce an effective antibody response to this epitope. The results also suggest that the 1KIG-based nanoparticle design may provide a promising N332-focused immunogen, which can be used individually or as a prime antigen in a heterologous immunization strategy.

### Dissecting MPER-specific antibody responses.

A similar immunization strategy was adopted to examine the MPER-directed antibody response in different structural contexts, as well as for comparison with the N332-directed response. Briefly, three groups of BALB/c mice were immunized with two bivalent MPER scaffolds—3G66_A_ES-Fc and 3O0P_A_ES-Fc—and the newly developed gp140.681.R1-1NOG trimer, with a fourth group included to test a trimer-prime/epitope-boost strategy. Since we did not observe any neutralization against tier 2 isolates (see Materials and Methods), we focused on serum binding analysis to dissect the early B-cell response.

**FIG 7:**
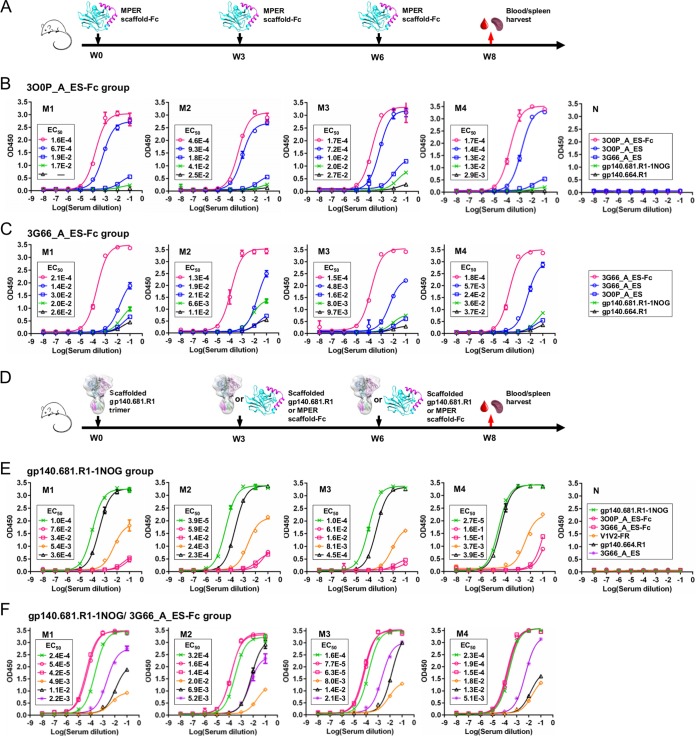
Analysis of the antibody response to MPER in mouse antisera. (A) Schematic view of the immunization regimen to test two bivalent MPER scaffolds in BALB/c mice. (B) ELISA binding of mouse antisera from the 3O0P_A_ES-Fc group to 2 monomeric MPER scaffolds, the Fc-fused MPER scaffold that was injected, the gp140.681.R1-1NOG trimer, and the parent gp140.664.R1 trimer. (C) ELISA binding of mouse antisera from the 3G66_A_ES-Fc group to the same set of antigens as described for panel B. (D) Schematic view of the immunization regimen to test the gp140.681.R1-1NOG trimer and a trimer-prime/epitope-boost strategy in BALB/c mice. (E) ELISA binding of mouse antisera from the trimer-alone group to the gp140.681.R1-1NOG trimer, the parent gp140.664.R1 trimer, two MPER-scaffold antigens, and a V1V2 nanoparticle (48). (F) ELISA binding of mouse antisera from the heterologous prime/boost group to the same set of antigens as described for panel E, with the inclusion of the monomeric version of the boost antigen (3G66_A_ES). The immunogens and the regimen details are labeled on the diagram for panels A and D. A naïve mouse sample is included in ELISA as a control, as shown in panels B and E. The EC_50_ values are labeled for all ELISA plots in panels B, C, E, and F, except for instances in which the highest OD_450_ value was below 0.1 or in the cases of ambiguous data fitting.

We first examined the antibody response to the grafted MPER epitope using antisera elicited by two bivalent scaffold antigens (Fig. 7A). Two MPER scaffolds—3O0P_A_ES and 3G66_A_ES—and their Fc-fusion proteins were utilized as coating antigens to detect the MPER-specific antibody response by ELISA (Fig. 7B and C). In contrast to our previous observation (Fig. 6B and S7A), mouse antisera elicited by one MPER-scaffold antigen showed weak binding to the other MPER scaffold, as indicated by a change in EC_50_ of up to ~100-fold, suggesting that the scaffold environment has a greater impact on epitope presentation for MPER. Furthermore, mouse antisera showed limited recognition of the gp140.681.R1-1NOG trimer, in contrast to the prominent trimer cross-reactivity observed for the two N332 nanoparticle groups. This suggests that different MPER conformations may be presented by the gp140.681.R1-1NOG trimer and the heterologous scaffolds. This notion is supported by data from diverse MPER structures in complex with NAbs such as 2F5 (74, 75), Z13 (76), and m66/m66.6 (77). However, other mechanisms such as immune tolerance (78) may also be involved in the diminished response to MPER. We then examined the scaffold-directed response in mouse antisera using two naked scaffolds (3O0P_A and 3G66_A), which showed negligible binding to bNAb 10E8 (Fig. S8B). Consistently, the mouse antisera exhibited stronger binding to the MPER scaffolds than to the naked scaffolds, as indicated by 10- to 100-fold changes in the EC_50_ (Fig. S8B), suggesting that a highly epitope-specific response was elicited by the bivalent MPER scaffolds.

We next examined the MPER-specific antibody response in the trimer group and the trimer-prime/epitope-boost group (Fig. 7D). The gp140.681.R1-1NOG trimer appeared to elicit a strong antibody response, as indicated by serum binding to this trimer and its parent construct (Fig. 7E). To better understand the immune recognition of this newly designed trimer construct, we utilized a V1V2 nanoparticle (48) to probe the apex-specific antibody response. Remarkably, mouse antisera elicited by a clade A trimer could recognize a clade C V1V2 nanoparticle across subjects, indicating that the apex is more stable in the gp140.681.R1-1NOG trimer than in its parent gp140.664.R1 trimer (Fig. 7E). As expected, no antigen binding was detected for the naïve mouse sample. Next, we tested antisera from the heterologous trimer-prime/epitope-boost group (Fig. 7F). All subjects in this group showed comparable antibody responses to both prime and boost antigens, in contrast to the differential binding observed for the N332 supersite using a similar regimen (Fig. 6F). Surprisingly, serum binding to 3G66_A_ES-Fc and 3G66_A_ES in this heterologous prime/boost group was greater than observed in the 3G66_A_ES-Fc group, as indicated by up to 10-fold differences in EC_50_ values (Fig. 7C and F), suggesting that priming the immune system with a full-length gp140 trimer enhances the antibody response to MPER. In addition, one injection of the gp140.681.R1-1NOG trimer elicited a more visible V1V2 apex-directed response than three injections of the gp140.664.R1 trimer (Fig. 6E). This suggests that the inclusion of MPER and a TS domain mimicking the transmembrane region in a membrane-bound gp160 Env spike (79) improves the immune recognition of a stabilized apex.

Overall, our results confirmed the utility of heterologous protein scaffolds and scaffolded gp140 trimers for presenting the full-length MPER in somewhat different conformations. Of note, the prominent apex-directed antibody response elicited by the gp140.681.R1-1NOG trimer may have important implications: similar trimer designs would present the complete antigenic surface of native Env by inclusion of MPER and a TS domain, which could also stabilize the apex and other bNAb targets. However, a more in-depth *in vivo* investigation is required to evaluate the full potential of these novel trimer constructs as vaccine candidates.

### Comparing splenic B-cell repertoire responses.

Spleen is a secondary lymphatic organ (SLO) that plays a critical role in the development of antigen-specific B cells (80). Here, we probed the repertoires of splenic B cells after immunization with various HIV-1 antigens. Briefly, mRNA extracted from splenic B cells of 8 immunized mice (1 from each group) and a naïve subject were used for antibody library preparation, in which 5′-rapid amplification of cDNA ends (RACE) PCR and mouse immunoglobulin (Ig) reverse primers were utilized to capture splenic B-cell repertoires in an unbiased manner (81–83). Next-generation sequencing (NGS) yielded a total of ~3.5 million reads for 9 mouse antibody libraries, each containing 146,801 to 548,871 reads. A primary analysis confirmed that the sequenced splenic B cells were predominantly IgMs (Table S2). A newly developed mouse antibodyomics pipeline (84) was then used to derive repertoire profiles for germline gene usage, degree of somatic hypermutation (SHM), and CDR3 loop length.

10.1128/mBio.00036-17.10 TABLE S2  Deep sequencing analysis of mouse splenic B-cell repertoires. Download TABLE S2, DOCX file, 0.02 MB. Copyright © 2017 Morris et al. 2017 Morris et al. This content is distributed under the terms of the Creative Commons Attribution 4.0 International license.

**FIG 8:**
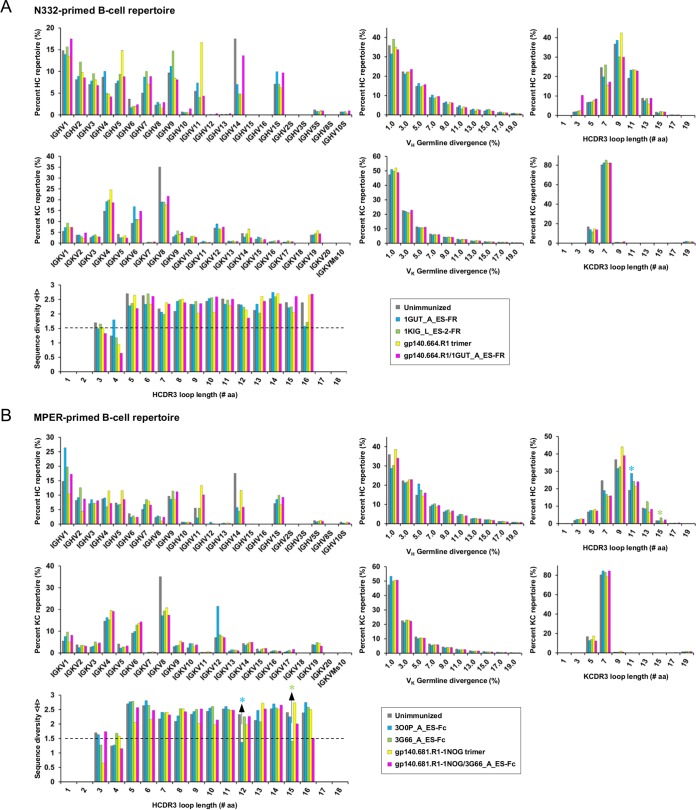
Deep sequencing analysis of mouse splenic B-cell repertoires. (A) N332-primed repertoires. (B) MPER-primed repertoires. Distributions are plotted for germline gene usage, germline gene divergence, or degree of SHM, CDR3 loop length, and sequence diversity measured by average Shannon entropy <H>. An empirical cutoff (1.5) used in the Shannon entropy analysis is indicated by a black dashed line, with the two HCDR3 groups showing <H> values below the cutoff labeled by asterisks. The corresponding peaks in the HCDR3 loop length distribution are labeled accordingly. The repertoire obtained from an unimmunized mouse is included in both panel A and panel B for comparison.

We first examined the B-cell repertoires activated by N332-focused antigens (Fig. 8A). A subset of germline genes were predominantly used in the unimmunized mouse repertoire, with IGHV1, IGHV14, and IGKV8 accounting for 15%, 17%, and 35% of their respective heavy-chain (HC) and κ chain (KC) repertoires. In contrast, the immunized mice displayed differential patterns of germline gene usage. While the nanoparticle-primed repertoires showed similar distributions, the trimer-activated B cells exhibited a broader germline gene usage with increased IGHV5 and IGHV11 frequencies, supporting the notion that diverse immunodominant epitopes are present on the Env trimer (73). Interestingly, the repertoire primed by gp140.664.R1 and boosted by 1GUT_A_ES-FR displayed an overall pattern similar to that of the unimmunized repertoire, with increased frequencies observed for specific germline genes (e.g., IGHVS1 and IGKV6) likely activated by 1GUT_A_ES-FR. In terms of SHM, the unimmunized repertoire exhibited average germline divergence values of 4.4% and 3.5% for HC and KC repertoires, respectively. In comparison, the four immunized mice showed similar SHM distributions, with a minor (4.8% on average) increase in germline divergence observed for the 1GUT_A_ES-FR-primed HC repertoire. In terms of CDR3 length, the five subjects displayed distinct patterns of distribution. The unimmunized mouse repertoire yielded an average HCDR3 length of 9.3 aa, whereas all antigen-activated repertoires contained more antibodies with 11-aa HCDR3 loops, which are ~10 aa shorter than those of N332-dependent bNAbs (60) and 2 to 3 aa shorter than the average HCDR3 length derived from normal human repertoires (50). Average Shannon entropy (85) was then calculated to assess the sequence diversity of HCDR3 loops ranging from 3 to 16 aa in length (see Materials and Methods). An average Shannon entropy of 1.5 or greater was observed for all HCDR3 groups with 5 to 16 aa for each subject, suggesting less effective B-cell activation.

We next examined the B-cell repertoires activated by MPER-focused antigens (Fig. 8B). The mouse repertoire primed by 3O0P_A_ES-Fc showed highly skewed usage of germline genes IGHV1 and IGKV12, which accounted for 26% and 22% of their respective HC and KC repertoires, whereas the one primed by 3G66_A_ES-Fc displayed a similar trend but primarily for V_H_ genes. Notably, the repertoire activated by gp140.681.R1-1NOG demonstrated more diverse usage of germline V_H_ genes than its parent gp140.664.R1 trimer, likely due to antibody responses directed to the MPER, TS domain, and trimer apex (Fig. 7E). The mouse repertoire primed by gp140.681.R1-1NOG and boosted by 3G66_A_ES-Fc displayed mixed germline gene usage, similar to the repertoire obtained from the trimer-prime/N332-boost regimen (Fig. 8A). The SHM distributions exhibited a consistent pattern for the B-cell repertoires primed by the two bivalent MPER scaffolds, with an average V_H_ germline divergence of ~4.8%. In terms of HCDR3 length, the two bivalent MPER scaffolds appeared to have elicited antibodies with longer HCDR3 loops ranging from 11 to 15 aa. Consistently, Shannon entropy analysis yielded a value below 1.5 for two HCDR3 groups with loop lengths of 12 and 15 aa, suggesting the expansion of specific antibody lineages within these HCDR3 groups.

Taken together, deep sequencing revealed the splenic B-cell responses to epitope- and trimer-based antigens at the repertoire level. In the context of heterologous protein scaffolds, MPER appeared to activate B cells more effectively than the N332 supersite, which is consistent with the hydrophobic peptide being a more visible target for immune recognition than a dense array of self-glycans. Although the native-like gp140 trimers did not demonstrate sufficient B-cell activation, they were capable of engaging diverse germline gene families.

## DISCUSSION

Identification of bNAbs, structural analysis of bNAb-antigen complexes, and immunogen design constitute the core components of a rational strategy for HIV-1 vaccine development (1–3). Significant efforts have been made to understand the structure, function, and evolution of bNAbs, providing a wealth of information to guide vaccine design (4, 8). Although epitope-focused vaccine design has been demonstrated for the early generation of HIV-1 NAbs (15–18, 20), the advent of a cleaved, soluble BG505 SOSIP.664 trimer with excellent mimicry of native Env (32–37) has renewed hope for an HIV-1 trimer vaccine (86). Recent progress in trimer platforms (43–46) and particulate display of gp140 trimers (47–49), as well as their assessment in rabbits and macaques (40, 87), has strengthened the foundation of trimer-based vaccine development. Therefore, epitope-focused and trimer-based design strategies represent distinct vaccine concepts that have been explored independently in the pursuit of an effective vaccine against HIV-1.

This study represented the first attempt to compare two vaccine design strategies with a focus on antibody responses to the N332 supersite near the trimer apex and MPER at the distal end of gp41_ECTO_, which are the targets of several best-in-class bNAbs (22, 60). Consistently, a recent study demonstrated that neutralizing antibodies to these two epitopes were associated with greater serum neutralization breadth and potency in a large patient cohort (88). However, a meaningful comparison of vaccine design strategies and epitope-specific antibody responses would require immunogens that have been optimized both structurally and antigenically. To this end, a scaffolding meta-server with broad coverage in database search (14, 50) was employed to identify heterologous protein scaffolds suitable for presenting these two distinct epitopes. Further considerations were given to multivalent epitope presentation (14) using various protein carriers, resulting in an array of epitope-focused immunogen designs. A new trimer platform derived from the analysis of HIV-1 metastability (46) has provided two native-like gp140 trimers, gp140.664.R1 and gp140.681.R1-1NOG, for comparison with N332- and MPER-focused immunogens, respectively. All design constructs have been thoroughly validated using biochemical, structural, and antigenic approaches, rendering a subset of highly optimized immunogen candidates for more in-depth *in vivo* evaluation.

We investigated the early, epitope-specific antibody response utilizing a mouse model and a short regimen. The BALB/c mouse strain was chosen on the basis of previous reports that this strain is prone to producing a stronger antigen-specific antibody response than the C57BL/6 strain (89, 90). For the N332 supersite, a cluster of self-glycans poses a significant challenge to the induction of an epitope-specific response. In natural infection, N332-dependent bNAbs may require 2 to 3 years to develop or involve immune escape at the N332 site (91, 92). As indicated by serum binding, two N332 nanoparticles elicited notable epitope-specific antibody responses that could also recognize the native-like gp140 trimer, whereas the trimer-elicited responses were diverted away from the N332 supersite and the trimer apex. Consistently, deep sequencing demonstrated that the trimer was capable of engaging diverse germline genes but did so with minimal impact on SHM and HCDR3 length. Thus, elicitation of antibodies against the N332 supersite remains a challenge, although priming the immune system with an N332 nanoparticle may prove to be beneficial. For the MPER epitope, the bivalent scaffold antigens and a scaffolded gp140.681 trimer appeared to elicit antibody responses that recognize different MPER conformations with limited cross-reactivity. Nonetheless, a heterologous prime/boost strategy was able to produce an antibody response with broader MPER reactivity, suggesting that such an immunization strategy may be more effective for this hydrophobic epitope. Surprisingly, a scaffolded gp140.681 trimer, but not its parent gp140.664 trimer, elicited a robust antibody response to the V1V2 apex even after the first injection. This suggests that engineering at the distal end of gp41_ECTO_ may have a significant impact on the structural stability and immune recognition of the quaternary trimer apex. As further demonstrated by repertoire analysis, the two bivalent MPER scaffolds appeared to be more immunogenic, showing highly skewed germline gene usage, increased SHM, and long HCDR3 loops indicative of B-cell lineage expansion upon antigen encounter.

Our report thus provides a set of promising epitope-focused immunogens for further *in vivo* evaluation. The scaffolded gp140.681 trimers may be superior to the current gp140.664 trimer platforms, as they can present all bNAb epitopes in native-like conformations with a more stable apex. Furthermore, the epitope-focused immunogens and native-like gp140 trimers can be combined in a heterologous immunization strategy, but such a regimen will require careful design and assessment. Deep sequencing of B-cell repertoires may provide further insights into antigen-induced B-cell events, which can benefit from purification of antigen-specific B cells and single-cell cloning. Overall, our work can serve as a useful template for rational vaccine development from structure-based design to quantitative B-cell response analysis (14).

## MATERIALS AND METHODS

### Epitope-scaffold design.

The computational procedure used for epitope scaffolding has been described previously (14, 50). In brief, a scaffolding meta-server was implemented in the same spirit as those meta-servers used in protein structure prediction. It combines results from six diverse structural alignment algorithms in scaffold search (50). To minimize the scaffold-directed response, a parameter was specified to limit the scaffold size so that the epitope would account for >10% of the scaffold. For the N332 supersite, the coordinates of the V3 stems (residues 293 to 298 and 329 to 334) in the crystal structure (PDB identifier [ID]: 3TV3) (23) were provided as input for the scaffolding meta-server (50) using selection parameters consisting of a protein size of 30 to 100 aa, an epitope matching length of 10 aa or greater, a C_α_ RMSD cutoff of 1.5 Å, and an epitope exposure cutoff of 0.4. For MPER, the coordinates in chain E (positions 656 to 684) and chain F (positions 659 to 684) of the 10E8-bound crystal structure (PDB ID: 4G6F) (22) were provided as input for the scaffolding meta-server (50) using selection parameters consisting of a protein size of 50 to 250 aa, an epitope matching length of 25 aa, a C_α_ RMSD cutoff of 3.0 Å, and an epitope exposure cutoff of 0.4. The scaffolds identified by the meta-server were docked into their respective bNAb-epitope complexes, and those with clash scores greater than 0.0 and 10.0 for the N332 supersite and MPER, respectively, were removed from the database.

The remaining scaffolds were used to calculate a coverage matrix that lists the number of scaffolds identified by each algorithm and the overlap of any two algorithms. The scaffolds were ranked according to the number of algorithms (or votes) by which they were identified (see Fig. S1A, S5A, and S5C in the supplemental material). Based on these metrics, a subset of scaffolds was manually selected from the high-voting groups, with structural features such as scaffold shape, orientation of the epitope-matching region to the scaffold, and ligand binding at the epitope-matching region visually inspected. Scaffolds of human origin or with other undesirable properties were removed. During epitope transplantation, the bNAb-interacting residues were grafted onto the epitope-matching region of a scaffold. Unfavorable interactions between the graft and the scaffold were then minimized by introducing mutations to the scaffold. During the design stage, structural inspection of the scaffolds may lead to alternative design ideas and thus additional constructs for experimental validation. For example, 3BN0_A contains exposed β-hairpins at both the N and C termini that can be utilized to present the N332 supersite, while 1KIG_L has a 12-aa flexible loop at the C terminus that may be truncated to reduce scaffold flexibility. As a result, two design variants were proposed for each scaffold and advanced to experimental validation. Thus, manual inspection plays a critical role in the final design stage, although the meta-server and consensus analysis can automate scaffold identification.

A stand-alone version of the scaffolding meta-server program, implemented as a Perl script, can be obtained from L. He and J. Zhu upon request.

### Protein expression and purification.

Epitope scaffolds and gp140 trimers were transiently expressed in HEK-293 F cells (Life Technologies, Inc., CA). Briefly, HEK-293 F cells were thawed and incubated with FreeStyle 293 expression medium (Life Technologies, Inc., CA) in a shaker incubator at 37°C, 120 rpm, and 8% CO_2_. When the cells reached a density of 2.0 × 10^6^/ml, expression medium was added to reduce the cell density to 1.0 × 10^6^/ml for transfection with polyethyleneimine (PEI-Max) (Polysciences, Inc.). For 60-ml HEK-293 F expression of the N332-focused antigens, cell culture was incubated with 300 μg of kifunensine (Tocris Bioscience), an α-mannosidase inhibitor, for 1 h prior to transfection to ensure the addition of Man_8/9_ glycan to the N332 position. A mixture of 54 μg of plasmid DNA and 21 μg of pAdVAntage in 2 ml of Opti-MEM transfection medium (Life Technologies, Inc., CA) was combined with 300 μl of PEI-Max (1.0 mg ml^−1^) in 2 ml of Opti-MEM. After incubation for 30 min, the DNA–PEI-Max complex was added dropwise to HEK-293 F cells. For 1-liter HEK-293 F expression of the N332-focused antigens, the amounts of kifunensine, DNA plasmids, Opti-MEM, and PEI-Max were scaled up proportionally. For MPER scaffolds, 1 liter of HEK-293 F cells was cotransfected with 900 μg of plasmid and 350 μg pAdVAntage diluted in 25 ml of Opti-MEM and combined with 5 ml of PEI-Max in 25 ml of Opti-MEM. For 1-liter transfection of gp140 trimers, 800 μg of plasmid, 300 μg of furin plasmid, and 300 μg of pAdVAntage were mixed into 25 ml of Opti-MEM and added to 25 ml of Opti-MEM with 5 ml of PEI-Max. After incubation for 30 min, the DNA–PEI-Max complex was added to 1 liter of HEK293F cells. Culture supernatants were harvested 5 days after transfection, clarified by centrifugation at 1,800 rpm for 22 min, and filtered using 0.45-μm-pore-size filters (Thermo Scientific). The epitope-focused and trimer antigens were also purified using different affinity resins. His-tagged, monomeric N332 scaffolds and MPER scaffolds were extracted from the supernatants using an immobilized Ni Sepharose excel column (GE Healthcare) and eluted with 500 mM imidazole. Fc-fused MPER scaffolds were extracted from the supernatants using a protein A Sepharose (PA) column (Vector Labs) and eluted with 0.2 M citric acid (pH = 3) in three 5-ml fractions that were each immediately neutralized using 400 μl of 2 M Tris base and concentrated to ~1 mg/ml. For N332 nanoparticles and gp140 trimers, a *Galanthus nivalis* lectin (GNL) column (Vector Labs) was used to extract proteins from the supernatants and eluted with phosphate-buffered saline (PBS) containing 500 mM NaCl and 1 M methyl-α-d-mannopyranoside. N332 nanoparticles and gp140 trimers were further purified using size exclusion chromatography (SEC) on a Superose 6 10/300 GL column and a Superdex 200 10/300 GL column (GE Healthcare), respectively. Protein concentrations were determined using a NanoDrop 8000 spectrophotometer (Thermo Scientific) and UV absorbance at 280 nm (UV_280_) with theoretical extinction coefficients.

### Cleavage of Fc from MPER scaffolds.

MPER scaffolds were cleaved from Fc using a four-step protocol: initial purification of Fc-fusion protein from HEK-293 F cells, Fc cleavage, protein A selection to remove Fc, and nickel (Promega) selection to remove the cleavage enzyme. Briefly, Fc-fusion protein was transiently transfected and purified by the use of protein A Sepharose beads as previously described. The desired amount of Fc scaffold was first mixed with ProTEV Plus enzyme (Promega) as recommended by the manufacturer. After incubation for approximately 12 to 24 h at 4°C, the cleavage reaction mixture was added to an Eppendorf tube containing preequilibrated protein A beads that was left shaking gently overnight (approximately 12 to 24 h) at 4°C. On the following day, this mixture was transferred into a poly-Prep chromatography column (Bio-Rad). The flowthrough, containing the MPER scaffold and ProTEV Plus enzyme, was collected and the beads were washed once with 10 ml of Tris-buffered saline (TBS) to obtain maximum recovery of the MPER scaffold. The protein A flowthrough and wash were then combined and concentrated with a buffer exchange to TBS for ELISA and nickel bead selection. Next, the concentrated protein A mixture was added to a 10-ml poly-Prep chromatography column containing preequilibrated nickel beads and was left shaking gently overnight (approximately 12 to 24 h) at 4°C. On the following day, the nickel bead flowthrough containing MPER scaffold only was collected and the beads were washed once with 10 ml of TBS to obtain maximum recovery of the antigen. The nickel bead flowthrough and wash were combined and concentrated with a buffer exchange to TBS for ELISA.

### SDS-PAGE and blue native (BN)-PAGE.

Designed antigens were analyzed by sodium dodecyl sulfate-polyacrylamide gel electrophoresis (SDS-PAGE) and blue native-polyacrylamide gel electrophoresis (BN-PAGE). The protein samples were mixed with loading dye and added to either a 10% Tris-glycine gel (Bio-Rad) or a 4 to 12% bis-Tris NuPAGE gel (Life Technologies, Inc.). For SDS-PAGE under reducing conditions, the antigen samples were first treated with dithiothreitol (DTT) (25 mM) and boiled for 5 min at 100°C. SDS-PAGE gels were run for 20 min at 250 V using SDS running buffer (Bio-Rad), while BN-PAGE gels were run for 2.5 h at 150 V using native PAGE running buffer (Life Technologies, Inc.) according to the manufacturer’s instructions. The gels were stained using Coomassie brilliant blue R-250 (Bio-Rad) and destained using a solution of 6% ethanol and 3% glacial acetic acid.

### Western blot analysis of N332 scaffolds.

SDS-PAGE was performed under reducing conditions as previously described using a total of 4 μg antigen for subsequent Western blot analysis. After gel electrophoresis, the resolved N332 scaffolds (reducing conditions) were transferred to a polyvinylidene difluoride (PVDF) membrane by the use of a Trans-Blot Turbo transfer system (Bio-Rad). The membrane was blocked with 5% nonfat milk. The immobilized N332 scaffolds were detected with 6×His epitope mouse antibody (Thermo Fisher) at 1 μg/ml and PGT128 human IgG at 4 μg/ml, with IRDye 680RD anti-mouse IgG and IRDye 800CW goat anti-human IgG (LI-COR Biosciences) (diluted 1:10,000) used as secondary antibodies, respectively. The immunoblots were analyzed with an Odyssey Infrared Imaging System and Image Studio software (Li-COR Biosciences).

### Enzyme-linked immunosorbent assay (ELISA).

Each well of a Costar 96-well assay plate (Corning) was first coated with 50 µl PBS containing 0.2 μg of the appropriate antigens. The plates were incubated overnight at 4°C and then washed five times with PBS containing 0.05% Tween 20. Each well was then coated with 150 µl of a blocking buffer consisting of PBS, 20 mg ml^−1^ blotting-grade blocker (Bio-Rad), and 5% fetal bovine serum (FBS). The plates were incubated with the blocking buffer for 1 h at room temperature and then washed 5 times with PBS containing 0.05% Tween 20. For antigen binding, the N332-dependent bNAbs (for the N332 supersite) or 10E8, 4E10, and two control bNAbs (for MPER) were diluted in the blocking buffer to a maximum concentration of 2 μg ml^−1^, followed by a 5-fold dilution series. For each bNAb dilution, a total of 50-μl volume was added to the appropriate wells. Each plate was incubated for 1 h at room temperature and then washed 5 times with PBS containing 0.05% Tween 20. For monomeric epitope scaffolds and N332 nanoparticles, a 1:5,000 dilution of goat anti-human IgG antibody (Jackson ImmunoResearch Laboratories, Inc.) was then made in the wash buffer (PBS containing 0.05% Tween 20), with 50 μl of this diluted secondary antibody added to each well. For Fc-fused MPER scaffolds, a 1:2,000 dilution of goat anti-human IgG F(ab')2 antibody (Abcam, Inc.) was made in the wash buffer (PBS containing 0.05% Tween 20), with 50 μl of this diluted secondary antibody added to each well. The plates were incubated with the secondary antibody for 1 h at room temperature and then washed 5 times with PBS containing 0.05% Tween 20. Finally, the wells were developed with 50 μl of TMB (Life Sciences) for 3 to 5 min before the reaction was stopped with 50 μl of 2 N sulfuric acid. The resulting plate readouts were measured at a wavelength of 450 nm.

Serum binding was conducted using a slightly modified ELISA protocol. Following incubation of each plate in blocking buffer, the mouse antisera were initially diluted by a factor of 10 in blocking buffer, followed by a 10-fold dilution series. A 50-μl volume of each dilution was then added to the appropriate wells. After incubation for 1 h and 5 washes, a 1:2,000 dilution of goat anti-mouse IgG antibody (Life Technologies, Inc.) was then prepared in the wash buffer (PBS containing 0.05% Tween 20), with 50 μl of this diluted secondary antibody added to each well. All plates were incubated for 1 h before being developed and measured at 450 nm.

### Octet binding assays.

The antibody binding kinetics of designed HIV-1 antigens were measured using an Octet Red96 instrument (FortéBio). All assays were performed with agitation set to 1,000 rpm in FortéBio 1× kinetic buffer. The final volume for all the solutions was 200 μl/well. Assays were performed at 30°C in solid black 96-well plates (Geiger Bio-One). Then, 1 μg/ml of protein–1× kinetic buffer was used to load the HIV-1 antibody on the surface of anti-human Fc capture biosensors (AHC) for 300 s. Typical capture levels were between 0.5 and 1 nm, and the variability within a row of eight tips did not exceed 0.1 nm. A 60-s biosensor baseline step was applied prior to the analysis of the association of the antibody on the biosensor to the antigen in solution for 200 s. A 2-fold concentration gradient of antigen starting at 10 μg/ml was used in a titration series of six. The dissociation of the interaction was followed for 300 s. Correction of baseline drift was performed by subtracting the averaged shift recorded for a sensor loaded with HIV-1 antibody but not incubated with antigen or a sensor without HIV-1 antibody but incubated with antigen. Octet data were processed by the use of FortéBio data acquisition software v.8.1. Experimental data were fitted with the binding equations describing a 1:1 interaction. Local fitting of the data sets was performed to obtain the optimal results for different antigens. The *K*_*D*_ value was determined using the estimated response at equilibrium for each antigen concentration rather than the *k*_on_ and *k*_off_ values.

### Negative-stain electron microscopy (EM).

For the scaffolded BG505 gp140.681 trimers, a 3-µl aliquot containing ~0.01 mg/ml of the sample was applied for 15 s onto a carbon-coated 400 Cu mesh grid that had been glow discharged at 20 mA for 30 s and then negatively stained with 2% uranyl formate for 45 s. Data were collected using a FEI Tecnai Spirit electron microscope operating at 120 kV, with an electron dose of ~30 e^−^/Å^2^ and a magnification of ×52,000 that resulted in a pixel size of 2.05 Å at the specimen plane. Images were acquired with a Tietz 4 k-by-4 k TemCam-F416 complementary metal-oxide semiconductor (CMOS) camera using a nominal defocus of 1,000 nm and the Leginon package. The scaffolded trimers were picked automatically using DoG Picker and put into a particle stack using the Appion software package. Reference-free, two-dimensional (2D) class averages were calculated using scaffolded trimers binned via the iterative multivariate statistical analysis/multireference alignment (msa/mra) clustering 2D alignment and IMAGIC software systems and sorted into classes. For the N332 nanoparticles, the sample preparation and data collection were the same as previously described (48).

### Mouse immunization.

Six-week-old female BALB/c mice were purchased from The Jackson Laboratory. The mice were housed in ventilated cages in environmentally controlled rooms at the Scripps Research Institute (TSRI), in compliance with an approved IACUC protocol and AAALAC guidelines. At week 0, each mouse was immunized with 100 μl (50 μg) of antigen formulated in 50 μl AddaVax adjuvant (InvivoGen) per the instructions of the manufacturer via the subcutaneous route. At week 3 and week 6, the animals were boosted with 10 μg of antigen formulated in AddaVax adjuvant. At week 8, the animals were terminally bled by cardiac puncture and the anticoagulant acid citrate dextrose (Sigma-Aldrich) was added to the samples at a 1:10 ratio. Samples were spun at 1,000 rpm for 10 min at 4°C to separate plasma and cells. Red blood cell lysis buffer (BioLegend) was added to the cell fraction. After 2 rounds of washing with PBS, peripheral blood mononuclear cells (PBMCs) were resuspended in Bambanker freezing media (Lymphotec Inc.). Spleens were also harvested and ground against a 40-μm-pore-size cell strainer (BD Falcon) to release the splenocytes into a cell suspension. The cells were centrifuged, treated with 10 ml of red blood cell (RBC) lysis buffer per manufacturer specifications, and resuspended in Bambanker freezing media for cell freezing.

### Pseudovirus production and neutralization assays.

Pseudoviruses were generated by transfection of 293T cells with an HIV-1 Env-expressing plasmid and an Env-deﬁcient genomic backbone plasmid (pSG3ΔEnv), as described previously (93). Pseudoviruses were harvested 72 h posttransfection for use in neutralization assays. Neutralizing activity was assessed using a single round of replication pseudovirus assay and TZM-bl target cells, as described previously (93). Briefly, TZM-bl cells were seeded in a 96-well flat-bottom plate. To this plate was added pseudovirus, which was preincubated with serial dilutions of mouse antisera for 1 h at 37°C. Luciferase reporter gene expression was quantified 72 h after infection upon lysis and addition of Bright-Glo luciferase substrate (Promega). To determine IC_50_ values, dose-response curves were fitted by nonlinear regression.

### Mouse B-cell library preparation and repertoire sequencing.

The 5′-RACE protocol was modified to improve the template preparation. Briefly, total RNA (including mRNA) was extracted from 10 to 20 million splenocytes into 30 μl of water with an RNeasy minikit (Qiagen). For unbiased antibody repertoire analysis, 5′-RACE was performed with a SMARTer RACE cDNA amplification kit (Clontech). The immunoglobulin PCRs were set up with platinum *Taq* High-Fidelity DNA polymerase (Life Technologies, Inc.) in a total volume of 50 µl, with 5 μl of cDNA as the template, 1 μl of 5′-RACE primer, and 1 μl of 10 µM reverse primer. The 5′-RACE primer contained a Personal Genome Machine (PGM) P1 adaptor, while the reverse primer contained a PGM A adaptor. We adapted the mouse 3′-C_γ_1-3 and 3′-C_μ_ inner primers and the 3′-mC_κ_ outer primer as reverse primers for 5′-RACE PCR processing of the heavy chains and κ chains, respectively (83). PCR was performed for 25 cycles, and the expected PCR products (500 to 600 bp) were gel purified (Qiagen).

### Ion Torrent PGM sequencing of mouse antibody libraries.

The sequencing procedure for human and mouse antibody libraries has been previously described (81, 84, 94). Briefly, the mouse heavy-chain and κ chain libraries were quantitated using a Qubit 2.0 fluorometer with a Qubit double-stranded DNA (dsDNA) HS assay kit and then used at a ratio of 1:1 for all the PGM sequencing experiments. The dilution factor required for Ion Torrent PGM template preparation was determined such that the final concentration was 50 pM. Template preparation was performed with an isothermal amplification (IA) kit (Thermo Fisher). Quality control of the template was determined by the use of a Qubit 2.0 fluorometer with an Ion Sphere quality control kit. Sequencing was performed on an Ion Torrent Personal Genome Machine (PGM) with a PGM Hi-Q 400 kit using an Ion 316 v2 chip for a total of 1,100 nucleotide flows. Raw data were processed without the 3′-end trimming during base calling in order to extend the read length.

### Bioinformatics analysis of antibody sequencing data.

The human antibodyomics pipeline (68, 69, 81, 94–97) has been used as the basis for development of an equivalent method for mouse antibody repertoire analysis (84). Briefly, the mouse antibodyomics pipeline consists of 5 steps: (i) data cleaning and formatting, (ii) germline gene assignment, (iii) template-based error correction, (iv) calculation of sequence identities to the known antibodies, and (v) determination of CDR3 and variable domain boundaries. In this study, instead of using the whole germline gene database (84), we selected a subset of germline genes −74 V_H_ genes and 67 V_κ_ genes—for the multiple-sequence alignment (MSA) procedure (step 5). With minimal effect on data quality, this modification significantly reduced the computational cost required for processing mouse antibody repertoire data and yielded efficiency comparable to that of the human pipeline.

The IgG and IgM frequencies were determined by matching the 3′ end of each heavy chain in the mouse antibody repertoire to the 3′-C_γ_ and 3′-C_μ_ primers. Briefly, a 25-nucleotide segment at the 3′ end of each heavy chain was aligned to each of the 5 primers (C_γ_1, C_γ2b_, C_γ2c_, C_γ_3, and C_μ_) to calculate the sequence identity, which in turn was used to assign a specific IgG or IgM family. Due to the sequencing errors or truncations that occurred in the raw data processing, the alignment approach yielded a more complete coverage of the data set than the matching of exact primer sequences. The obtained IgG and IgM frequencies are listed in Table S2 in the supplemental material.

Given a group of HCDR3 loops with the same length, sequence diversity can be estimated as parameter <H>, which represents the Shannon entropy at each amino acid position averaged over the whole sequence (85). We have devised the following three-step procedure to calculate <H> from the antibody repertoire data: (i) cluster the nucleotide sequences of HCDR3 loops according to their lengths and discard any sequences with length not divisible by 3; (ii) translate the nucleotide sequences within each cluster into amino acid sequences and discard any sequences with stop codons; and (iii) given an HCDR3 length defined by the cluster, calculate the Shannon entropy at each amino acid position using the following formula (85) before taking the average:

$$H=\sum_{j=1}^{20}\frac{k_{j}}{n}log_{2}\frac{k_{j}}{n}\;\;\;\text{and}\;\;H=\frac{\sum_{i}^{N}H_{i}}{N}$$

where *k*_*j*_ is the frequency of residue type *j* at a particular amino acid position *i*, *n* is the number of HCDR3 sequences within a cluster, and *N* is the HCDR3 loop length. The value of parameter <H> represents the sequence diversity of HCDR3 loops with a specific length. A lower value of average entropy <H> would indicate that the HCDR3 loops within a cluster share a higher degree of sequence similarity.
