# Supplementary figures and images for: Differential Antibody Responses to Conserved HIV-1 Neutralizing Epitopes in the Context of Multivalent Scaffolds and Native-Like gp140 Trimers
Source: mBio. 2017 Feb 28;8(1):e00036-17. doi: 10.1128/mBio.00036-17 (PMC5347340; doi:10.1128/mBio.00036-17)

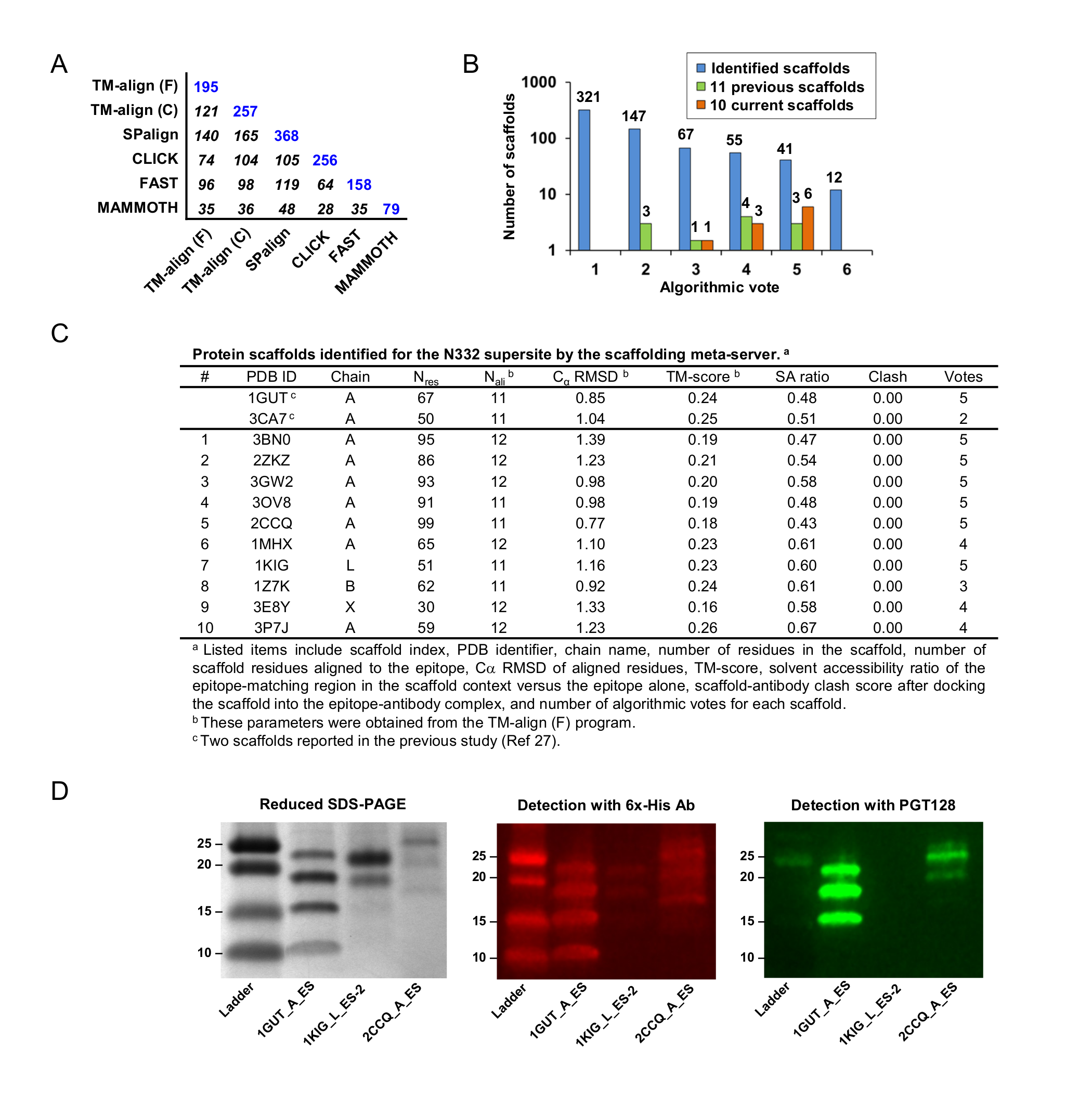

Supplement: FIG S1 [file mbo001173209sf1.tif]

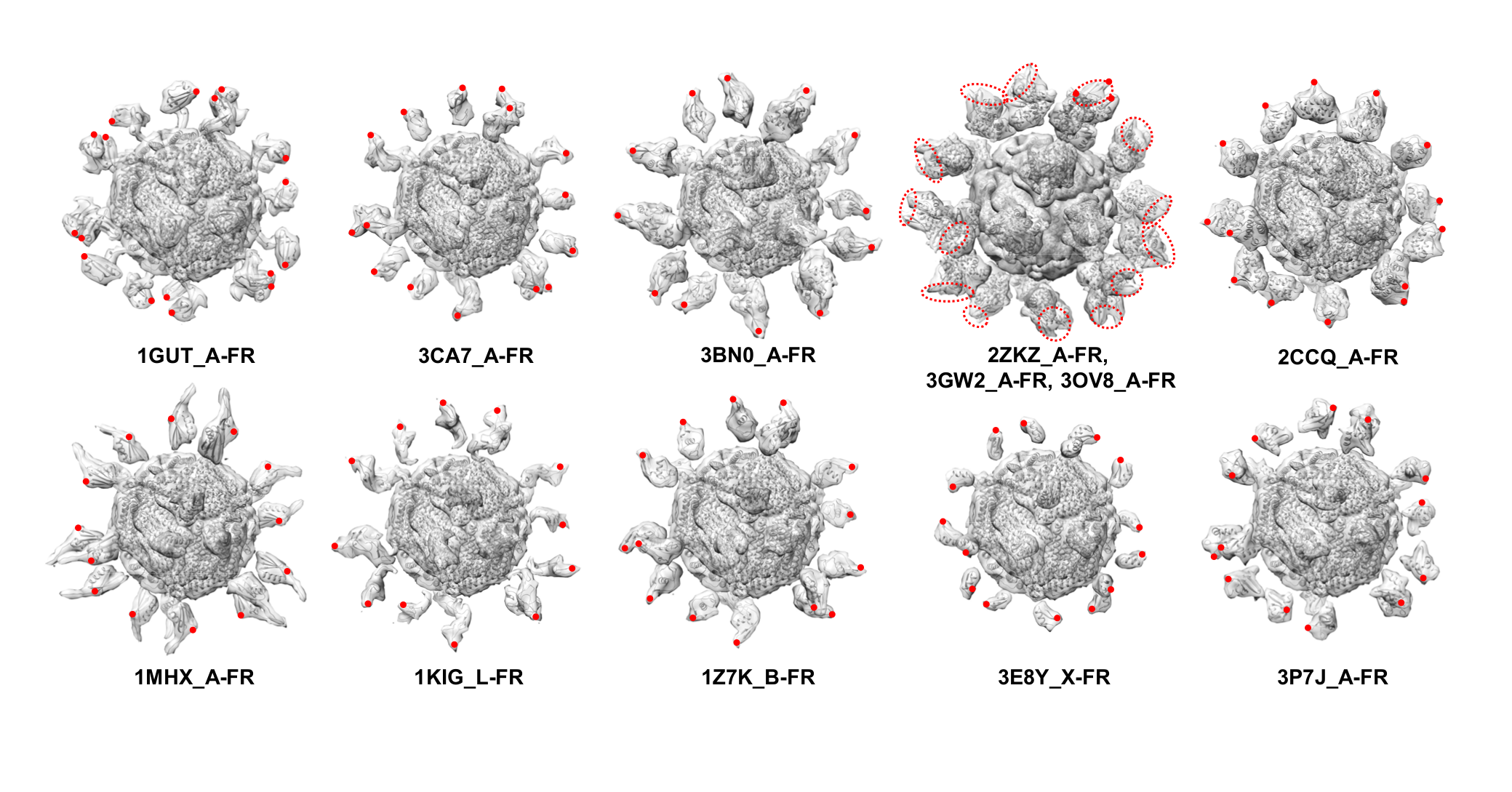

Supplement: FIG S2 [file mbo001173209sf2.tif]

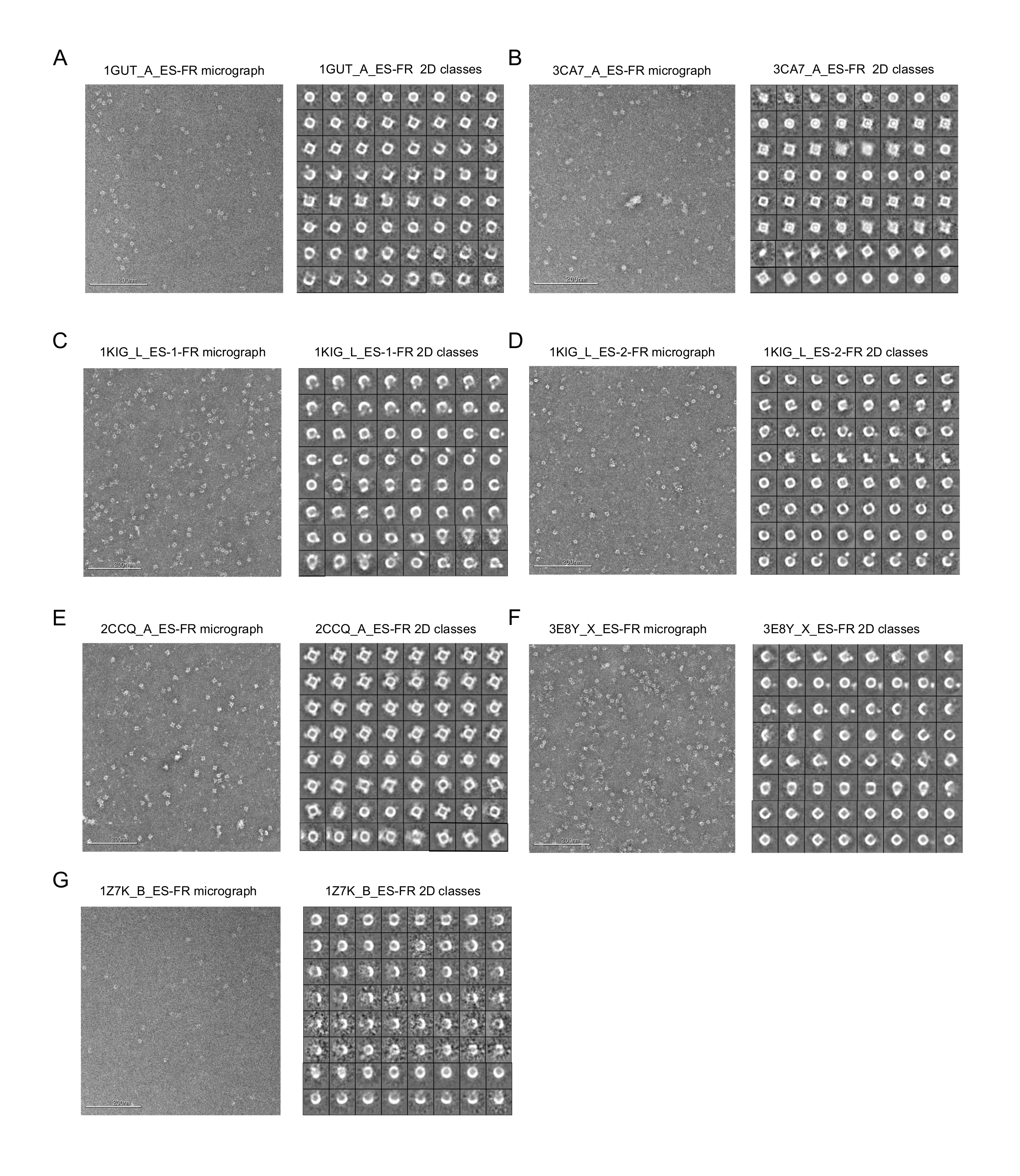

Supplement: FIG S3 [file mbo001173209sf3.tif]

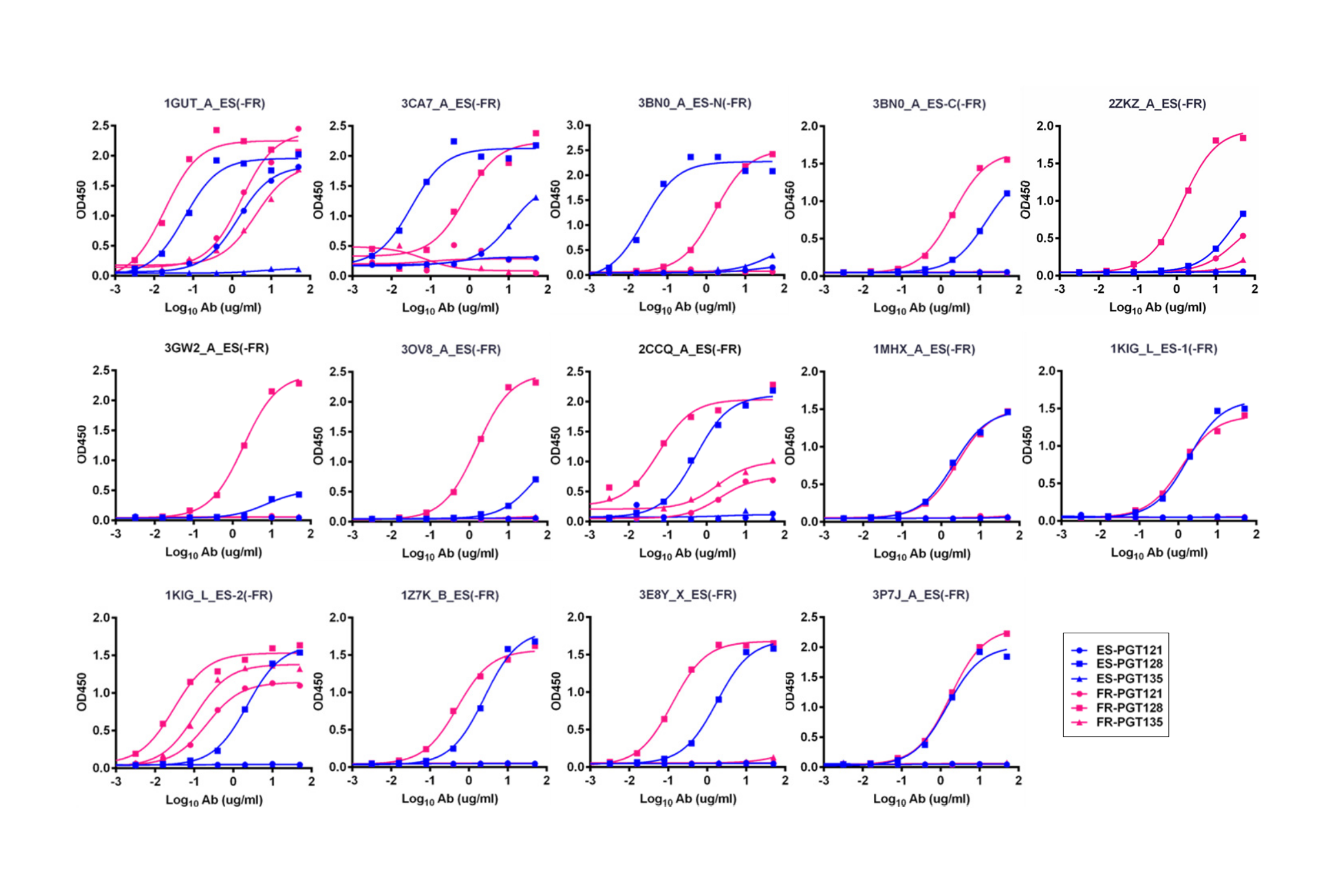

Supplement: FIG S4 [file mbo001173209sf4.tif]

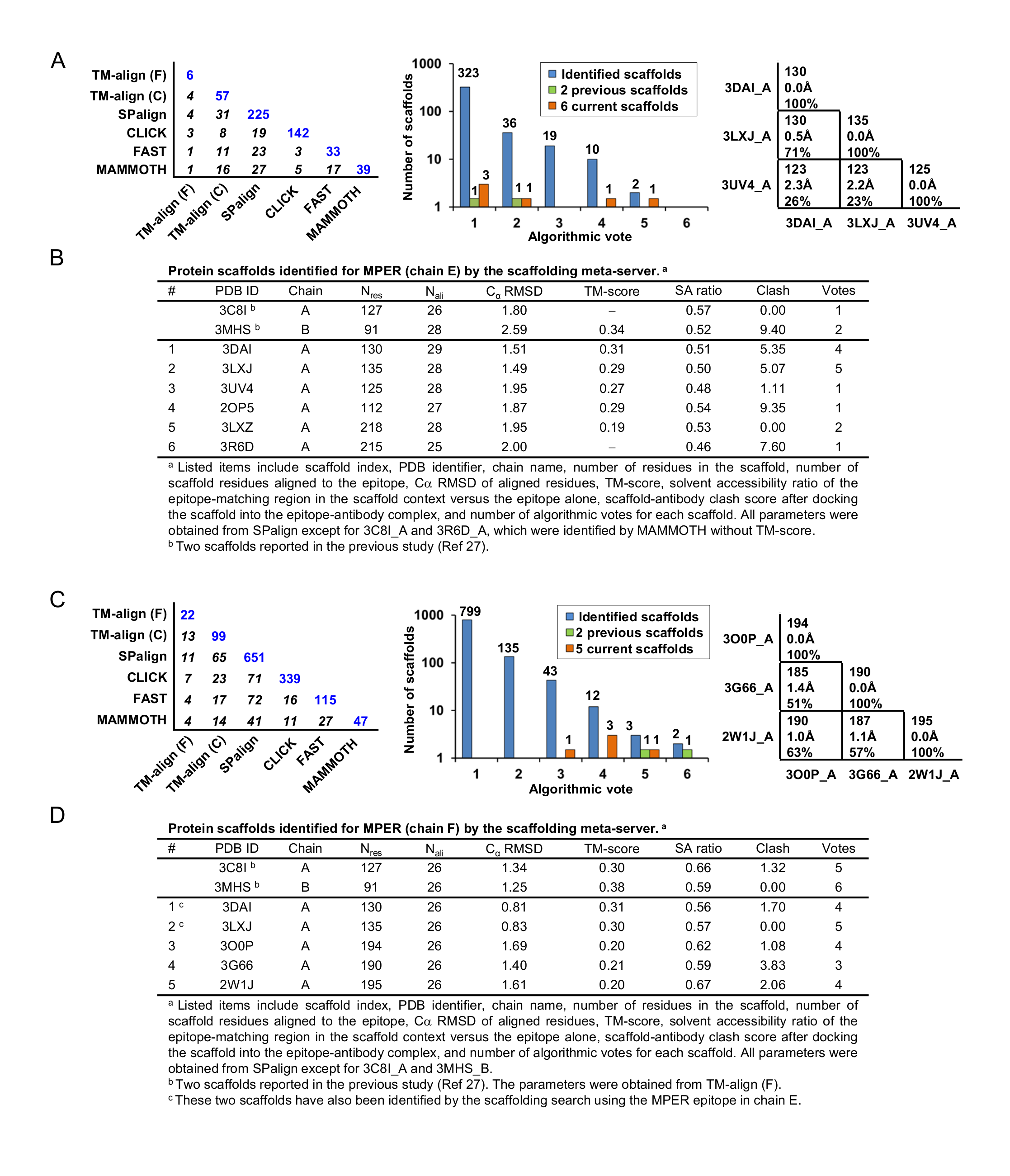

Supplement: FIG S5 [file mbo001173209sf5.tif]

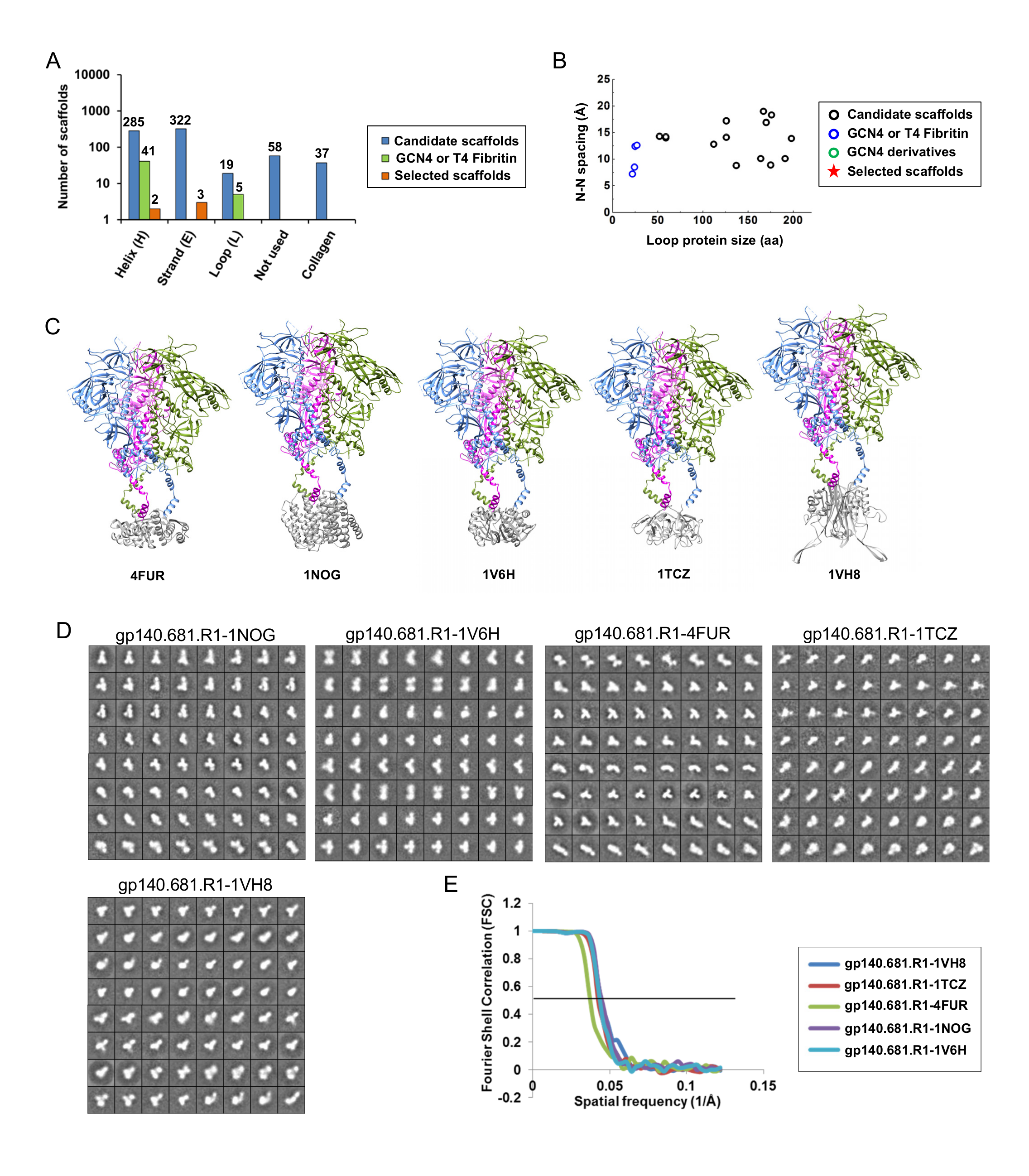

Supplement: FIG S6 [file mbo001173209sf6.tif]

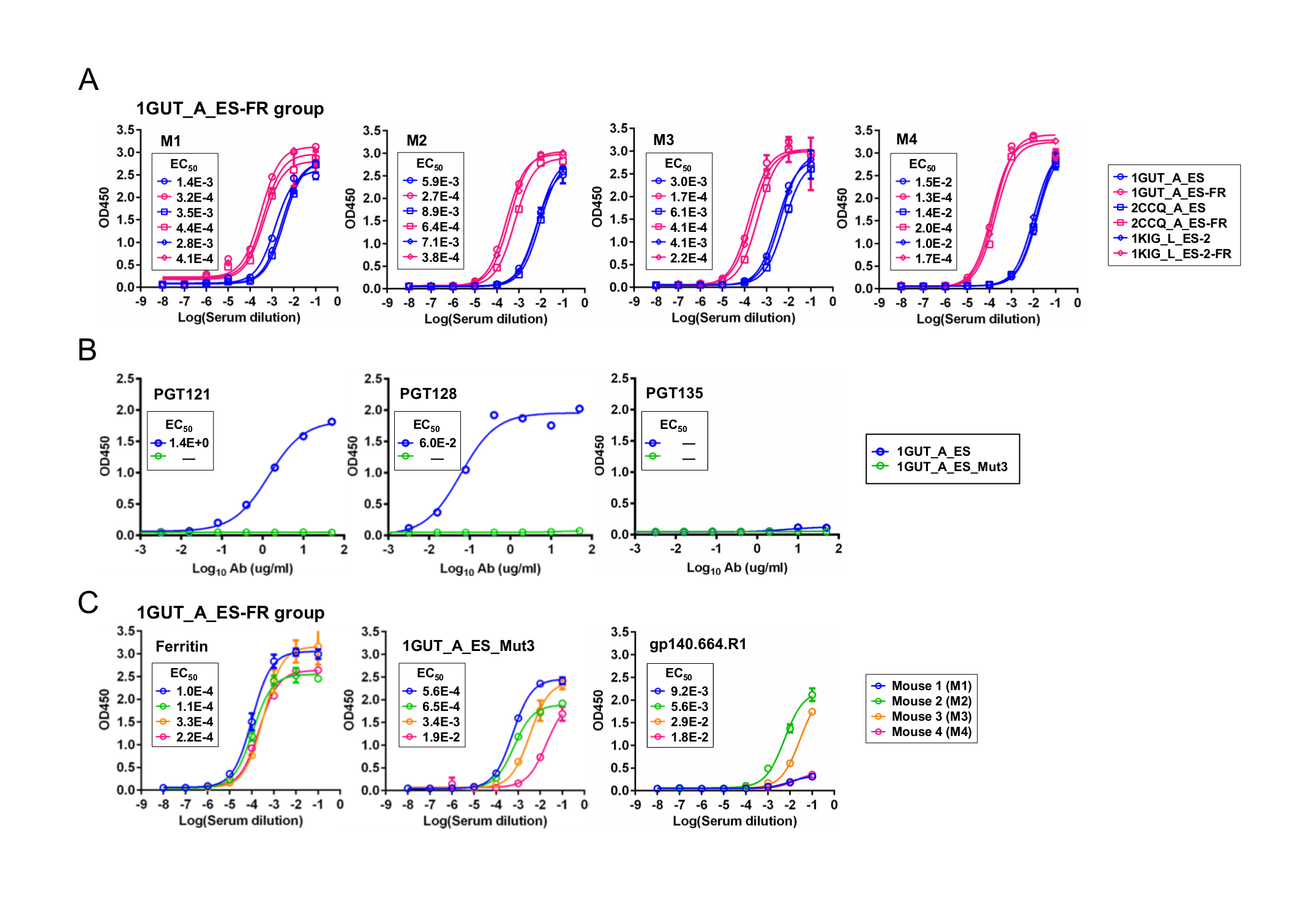

Supplement: FIG S7 [file mbo001173209sf7.tif]

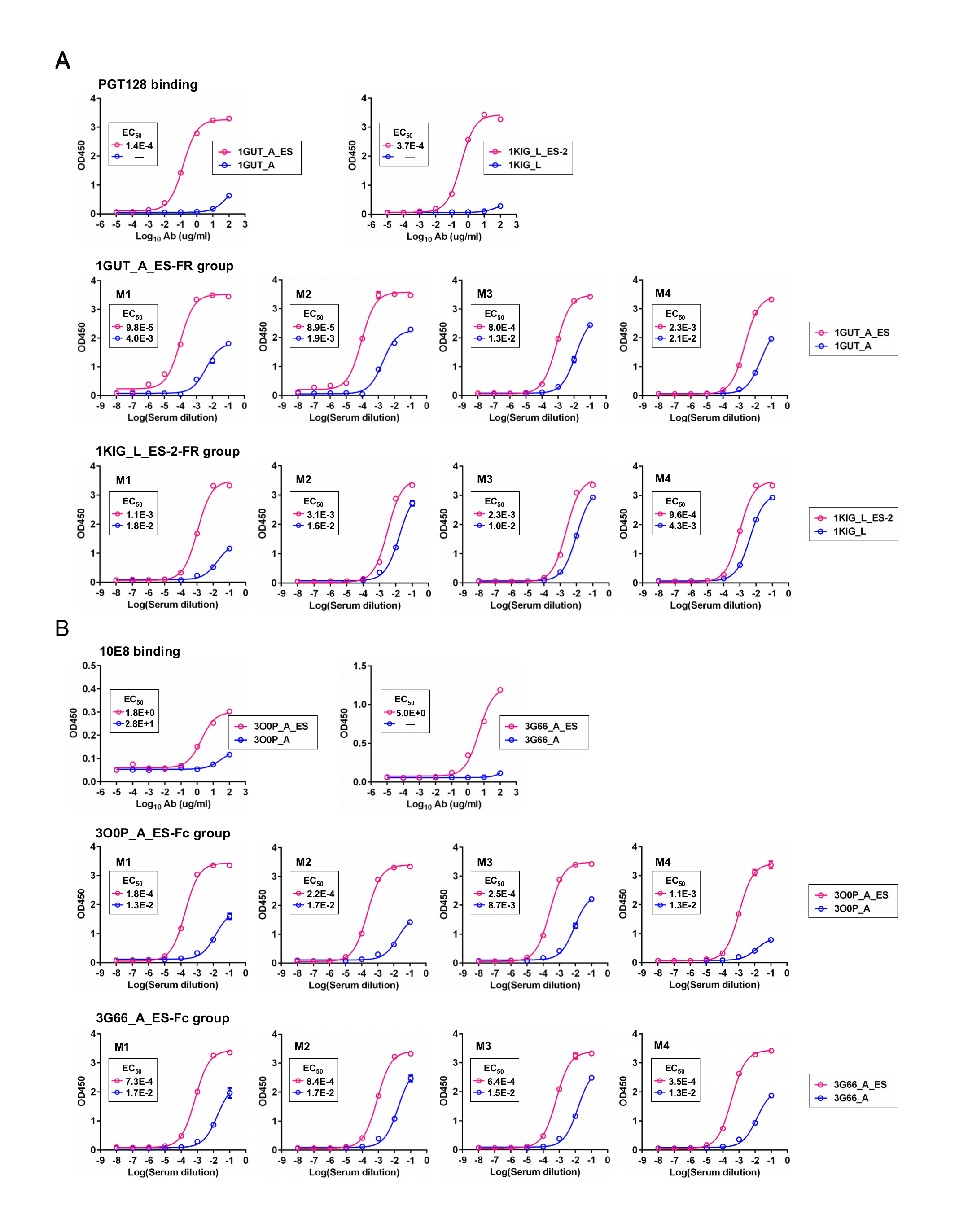

Supplement: FIG S8 [file mbo001173209sf8.tif]
