# Supplementary material for: Differential Antibody Responses to Conserved HIV-1 Neutralizing Epitopes in the Context of Multivalent Scaffolds and Native-Like gp140 Trimers
Source: mBio. 2017 Feb 28;8(1):e00036-17. doi: 10.1128/mBio.00036-17 (PMC5347340; doi:10.1128/mBio.00036-17)
Supplement: TABLE S1 [file mbo001173209st1.docx]

| **TABLE S1** Amino acid sequences of rationally designed HIV-1 antigens ^a^ | |
| --- | --- |
| Construct name | Amino acid sequence |
| Epitope-scaffolds and nanoparticle constructs designed for the N332 supersite | |
| 1GUT_A_ES | SISARNQLKGKVVGLNCTRPNNNTRPGEIIGDIRQAHCNV**T**LEIAGGNKITSIISLDSVEELGVKEGAELTAVVKSTDVMILA**GS**HHHHHH |
| 3CA7_A_ES | **A**T**A**PTYKCPETFDAWYCLNDAHC**EI**NCTRPNNNTRPGEIIGDIRQAHCN**I**SCECAIGFMGQRCEYKE**GS**HHHHHH |
| 3BN0_A_ES-N | AVRIRLAKNCTRPNNNTRPGEIIGDIRQAHCNY**S**IVVM**GSG**IDILGTYDPKRKVL**A**NVYPEKVKEWVLKGVELSHRAKAILWNHGILKEVVPEGYEMKRVGDYYVFEKRE**ASGS**HHHHHH |
| 3BN0_A_ES-C | AV**A**IRLAKFGRKHHPIYRIVVM**GSG**IDILGTYDPKRKVLINVYPEKVKEWVLKGV**A**LSHRAKAILWNHGILKEVVPEGYEMNCTRPNNNTRPGEIIGDIRQAHCNF**S**KRE**ASGS**HHHHHH |
| 2ZKZ_A_ES | YMSLEDDAELLKTMAHPMRLKIVNELYKHKAL**A**VTQIIQILKLPQSTVSQHL**A**KMRGKVLKRNCTRPNNNTRPGEIIGDIRQAHCNYSINNPKVEGIIKLLN**ASGS**HHHHHH |
| 3GW2_A_ES | RKAALLDQVARVGKALANGRRLQILDLLAQG**AG**AVEAIATATGM**A**LTTASANLQALKSGGLVEANCTRPNNNTRPGEIIGDIRQAHCNY**S**IAGEDVARLFALVQVVADE**ASGS**HHHHHH |
| 3OV8_A_ES | ERIKLLFKEKALEILMTIYYESLGGN**G**V**A**IQYIASKVNSPHSYVWLIIKKFEEAKMVE**A**NCTRPNNNTRPGEIIGDIRQAHCNI**S**LTDKGQKIAQQIKSIIDIMEND**ASGS**HHHHHH |
| 2CCQ_A_ES | GSASPAVAELCQNTPETFLEASKLLLTYADNILRNPND**A**KYRSIRIGNTAFSTRLLPVRGAVECLFEMGFNCTRPNNNTRPGEIIGDIRQAHCNF**SA**KASVEQLQKIRDLIAIER**ASGS**HHHHHH |
| 1MHX_A_ES | AMDTYKLFIVIGDRVVVVTTEAVDAATAEKVFKQYANDNGVDGEWN**Y**TRPNNNTRPGEIIGDIRQAH**F**NVTE**GS**HHHHHH |
| 1KIG_L_ES-1 | CSLDNGGCDQFCN**E**TRPNNNTRPGEIIGDIRQAH**V**NCSCAHGYVLGDDSKSCVSTERFPCGKFTQGR**GS**HHHHHH |
| 1KIG_L_ES-2 | CSLDNGGCDQFCN**E**TRPNNNTRPGEIIGDIRQAH**V**NCSCAHGYVLGDDSKSCVST**GS**HHHHHH |
| 1Z7K_B_ES | VPMDCSRY**EQ**NCTRPNNNTRPGEIIGDIRQAHCNI**S**CNKALNPVCGTDGVTYDNECVLCAHNLEQGTSVGKKHDGEC**GSGS**HHHHHH |
| 3E8Y_X_ES | ACYSSDCRVKCVAMGFS**E**GNCTRPNNNTRPGEIIGDIRQAHCNC**S**K**GS**HHHHHH |
| 3P7J_A_ES | TGFDRGLEAEKIL**E**ANCTRPNNNTRPGEIIGDIRQAHCNF**S**IQFKGVDQAE**A**VPSSVANEKIPRMVIHFYEERLS**GS**HHHHHH |
| Epitope-scaffold fused to ferritin subunit | **[N332EPITOPE_SCAFFOLD]ASGGGGS**DIIKLLNEQVNKEMQSSNLYMSMSSWCYTHSLDGAGLFLFDHAAEEYEHAKKLIIFLNENNVPVQLTSISAPEHKFEGLTQIFQKAYEHEQHISESINNIVDHAIKSKDHATFNFLQWYVAEQHEEEVLFKDILDKIELIGNENHGLYLADQYVKGIAKSRKS |
| Epitope-scaffolds and Fc-fusion constructs designed for MPER | |
| 3C8I_A_ES | DLVPAMIAEVNPRDMVVMALVNTNVDPTLPPRWALATRNITAIPGIEGDTRKVGTRIPAV  AVTGQRSVGNQDSWDQISPMPIAWATPD**LL**VI**DK**AES**LWNWF**QWT**N**L**LW**NL**R**KLDQVRET  KFDLLEL**GS**HHHHHH |
| 3MHS_B_ES | TAQ**A**KSQIQQYLVESGNYELISNELKARL**K**QEGW**Y**DKVKDLTKSEMNINE**A**TN**NE**QI**KLL**  VE**D**KAL**SLWNWF**TR**TN**VL**W**QIREFLEEIVDT**GS**HHHHHH |
| 3DAI_A_ES | SMQEEDTFRELRIFLRNVTHRLAIDKRFRVFTKPVDPDEVPDYVTVIKQPMDLSSVISKIDLHKYL**G**V**A**DYLRDIDLICSNALEYNPDRDPGDRLIRHRA**EQ**L**LLE**A**DK**I**ASLWNWF**D**ITN**L**LWI**I**RAGS**HHHHHH |
| 3LXJ_A_ES | SMEDQEENTLRELRLFLRDVTKRLATDKRFNIFSKPVDIEEVSDYLEVIKEPMDLSTVITKIDKHNYLTAKDFLKDIDLICSNALEYNPDKDPGDKIIRHRA**EQ**L**LLE**A**DK**I**ASLWNWFDITN**L**LWI**I**RAGS**HHHHHH |
| 3UV4_A_ES | SMDDDQVAFSFILDNIVTQKMMAVPDSWPFHHPVNKKFVPDYYKVIVNPMDLETIRKNISKHKY**GL**ESFLDDVNLILANSVKYNGPESQYTKTAQEIV**L**V**LDK**T**ASLWNWFDI**T**N**L**LWY**I**RAGS**HHHHHH |
| 2OP5_A_ES | TDETAFLNSLFMDFTSENELELFLKSLDEVWSEDLYSRLSAAGLIRHVISKVWNKEQHRISMVFEYDS**EQ**GY**LE**C**DK**II**SLWNWFD**L**TN**KL**WY**F**RG**FKIHNNRGVVVSEFIRS**GS**HHHHHH |
| 3LXZ_A_ES | SLKLYGFSVSNYYNMVKLALLEKGLTFEEVTFYGGQAPQALEVSPRGKVPVLETEHGFLSETSVILDYIEQTQGGKALLPADPFGQAKVRELLKEIELYIELPARTCYAESFFGMSVEPLIKEKARADLLAGFATLKRNGRFAPYVAGEQLTLADLMFCFSVDLANAVGKKVLNIDFLADF**E**QA**LL**LL**DK**M**ASLWNWFD**I**TNDLWY**S**RA**AFMEMIRSG**GS**HHHHHH |
| 3R6D_A_ES | SNAMYKYITILGAAGQIAQKLTATLLTYTDMHITLYGRQLKTRIPPEIIDHERVTVIEGSFQNPGKLEQAVTNAEVVFVGAMESGSDMASIVKALSRKNIRRVIGVSMAGLSGEFP**LLELDKIASLWNWF**Y**ITN**E**LW**A**IRA**LRESNLNYTILRLTWLYNDPEKTDYELIPEGAQFNDAQVSREAVVKAIFDILHAADETPFHRTSIGVGEPGTHYDKPSFH**GS**HHHHHH |
| 3O0P_A_ES | QTQDF**DK**AA**SLWNWF**EI**TN**RM**WY**A**R**AYNDSLNNVHLEDPYEKKR**S**G**S**GIAEYARMLEVSEKIGIISVPKIGQKLPIFAGSSQEVLSKGAGHLEGTSLPIGGNSTHTVITAHSGIPDKELFSNLKKLKKGDKFYIQNIKETIAYQVDQIKVVTPDNFSDLLVVPGHDYATLLTCTPIMVNTHRLLVRGHRIPYK**GS**HHHHHH |
| 3G66_A_ES | NEVI**L**EFD**K**TVS**LWNWF**EL**TN**R**LW**LA**R**AFNATLKPSEILDPFTEQEKKKGVSEYANMLKVHERIGYVEIPAIDQEIPMYVGTSEDILQKGAGLLEGASLPVG**GN**THTVITAHRGLPTAELFSQLDKMKKGDIFYLHVL**GN**VLAYQVDQIVTVEPNDFEPVLIQHGEDYATLLTCTPYMINSHRLLVRGKRIP**GS**HHHHHH |
| 2W1J_A_ES | QQIADFDK**W**K**SLWNWF**DI**TN**RM**W**LA**R**AFNDSLNNVVSGDPWSEEMKKKGRAEYARMLEIHERMGHVEIPVIDVDLPVYAGTAEEVLQQGAGHLEGTSLP**A**GG**G**STHAVITAHTGLPTAKMFTDLTKLKVGDKFYVHNI**GG**VMAYQVDQVKVIEPTNFDDLLIVPGHDYVTLLTCTPYMINTHRLLVRGHRIP**GS**HHHHHH |
| Epitope-scaffold fused to Fc domain ^b^ | **[MPEREPITOPE_SCAFFOLD]GSG**ENLYFQGKTHTCPPCPAPEAAGGPSVFLFPPKPKDTLMISRTPEVTCVVVDVSHEDPEVKFNWYVDGVEVHNAKTKPREEQYNSTYRVVSVLTVLHQDWLNGKEYKCKVSNKALPAPIEKTISKAKGQPREPQVYTLPPSRDELTKNQVSLTCLVKGFYPSDIAVEWESNGQPENNYKTTPPVLDSDGSFFLYSKLTVDKSRWQQGNVFSCSVMHEALHNHYTQKSLSLSPGK |
| Scaffolded BG505 gp140.681.R1 trimers ^c^ | |
| gp140.681.R1-TS | AENLWVTVYYGVPVWKDAETTLFCASDAKAYDTEKHNVWATHACVPTDPNPQEIHLENVTEEFNMWKNNMVEQMHTDIISLWDQSLKPCVKLTPLCVTLQCTNVTNNITDDMRGELKNCSFNMTTELRDKKQKVYSLFYRLDVVQINENQGNRSNNSNKEYRLINCNTSAITQACPKVSFEPIPIHYCAPAGFAILKCKDKKFNGTGPCPSVSTVQCTHGIKPVVSTQLLLNGSLAEEEVMIRSENITNNAKNILVQFNTPVQINCTRPNNNTRKSIRIGPGQAFYATGDIIGDIRQAHCNVSKATWNETLGKVVKQLRKHFGNNTIIRFANSSGGDLEVTTHSFNCGGEFFYCNTSGLFNSTWISNTSVQGSNSTGSNDSITLPCRIKQIINMWQRIGQAMYAPPIQGVIRCVSNITGLILTRDGGSTNSTTETFRPGGGDMRDNWRSELYKYKVVKIEPLGVAPTRCKRRVVGRRRRRRAVGIGAVFLGFLGAAGSTMGAASMTLTVQARNLLSGNPDWLPDMTVWGIKQLQARVLAVERYLRDQQLLGIWGCSGKLICCTNVPWNSSWSNRNLSEIWDNMTWLQWDKEISNYTQIIYGLLEESQNQQEKNEQDLLALDKWASLWNWFDITNWLWYIRA**AS[TS DOMAIN]** |
| TS_1_: 1NOG | SPVVEVQGTIDELNSFIGYALVLSRWDDIRNDLFRIQNDLFVLGEDVSTGGKGRTVTREMIDYLEARVKEMKAEIGKIELFVVPGGSVESASLHMARAVSRRLERRIVAASKLTEINKNVLIYANRLSSILFMHALISNKRLNIPEKIW |
| TS_2_: 1V6H | EEVVLITVPSEEVARTIAKALVEERLAACVNIVPGLTSIYRWQGEVVEDQELLLLVKTTTHAFPKLKERVKALHPYTVPEIVALPIAEGNREYLDWLRENTG |
| TS_3_: 4FUR | HLTPREFDKLVIHMLSDVALKRKNKGLKLNHPEAVAVLSAYVLDGAREGKTVEEVMDGARSVLKADDVMDGVPDLLPLIQVEAVFSDGSRLVSLHNPIT |
| TS_4_: 1TCZ | SDPAHTATAPGGLSAKAPAMTPLMLDTSSRKLVAWDGTTDGAAVGILAVAADQTSTTLTFYKSGTFRYEDVLWPEAASDETKKRTAFAGTAISIV |
| TS_5_: 1VH8 ^d^ | LIRIGHGFDVHAF**GSG**FIAHSDGDVALHALTDAILGAAALGDIGKLFP**NDT**NADSRGLLREAFRQVQEKGYKIGNVDITIIAQAPKMRPHIDAMRAKIAEDLQCDIEQVNVKATTTEKLGFTGRQEGIACEAVALLIRQ |
| ^a^ In epitope-focused antigen constructs, the epitope-matching region is shown in gray shade with the mutations and linkers (e.g. in ferritin- and Fc-fusion constructs) colored in magenta. All enzymatic sites (AS, GS, and ASGS) are colored in orange.  ^b^ In Fc-fusion constructs, the cleavage site recognized by tobacco etch virus (TEV) protease is shown in yellow shade.  ^c^ In the gp140.681.R1-TS constructs, the redesigned heptad region 1 (HR1) (Ref 46) is shown in cyan shade, with MPER shown in gray shade. Of note, an optimized N-terminal signal peptide for secretory expression (MDAMKRGLCCVLLLCGAVFVSPSQEIHARFRRGAR) has been used for gp140 trimer production.  ^d^ A protruding hairpin on the surface of 1VH8 structure (residues 14-31) that facilitates the assembly of a tetramer of 1VH8 trimers is replaced with a GSG linker (in magenta) to reduce potential aggregation of expressed gp140.681.R1-1VH8 trimers. An unstructured loop is shortened and replaced with an *N*-linked glycosylation site – NDT (in magenta) to further increase solubility and reduce aggregation. | |
