# Supplementary material for: Differential Antibody Responses to Conserved HIV-1 Neutralizing Epitopes in the Context of Multivalent Scaffolds and Native-Like gp140 Trimers
Source: mBio. 2017 Feb 28;8(1):e00036-17. doi: 10.1128/mBio.00036-17 (PMC5347340; doi:10.1128/mBio.00036-17)
Supplement: TABLE S2 [file mbo001173209st2.docx]

| **TABLE S2**  Deep sequencing analysis of mouse splenic B-cell repertoires | | | | | | | | | |
| --- | --- | --- | --- | --- | --- | --- | --- | --- | --- |
| Antibodyomics pipeline processing of mouse splenic B-cell sequencing data | | | | | | | | | |
| Library  index | Antigen | Mouse  index | N_read_ | Chain | N_chain_ | <Length> | N_full_ | N_usable_ | Perc_usable_ |
| 1 | 1GUT_A_ES-FR | M1 | 146,801 | H | 54,524 | 532.0 | 37,732 | 29,313 | 54% |
|  |  |  |  | K | 72,391 | 498.8 | 67,006 | 53,385 | 74% |
| 2 | 1KIG_L_ES-2-FR | M1 | 569,316 | H | 190,584 | 561.5 | 128,522 | 105,309 | 55% |
|  |  |  |  | K | 253,822 | 521.1 | 232,050 | 179,819 | 71% |
| 3 | gp140.664.R1 | M2 | 318,620 | H | 133,453 | 544.1 | 96,512 | 74,076 | 56% |
|  |  |  |  | K | 152,660 | 511.3 | 144,828 | 113,478 | 74% |
| 4 | gp140.664.R1/ | M2 | 323,346 | H | 72,623 | 517.4 | 48,243 | 38,107 | 52% |
|  | 1GUT_A_ES-FR |  |  | K | 217,144 | 506.2 | 204,135 | 160,209 | 74% |
| 5 | 3O0P_A_ES-Fc | M3 | 548,871 | H | 220,412 | 546.3 | 159,444 | 124,525 | 56% |
|  |  |  |  | K | 259,967 | 502.0 | 241,310 | 189,374 | 73% |
| 6 | 3G66_A_ES-Fc | M4 | 258,485 | H | 58,297 | 538.5 | 41,183 | 31,932 | 55% |
|  |  |  |  | K | 166,196 | 514.4 | 156,760 | 122,131 | 73% |
| 7 | gp140.681.R1-1NOG | M4 | 407,444 | H | 189,411 | 569.3 | 143,937 | 115,352 | 61% |
|  |  |  |  | K | 180,019 | 508.8 | 171,006 | 134,774 | 75% |
| 8 | gp140.681.R1-1NOG/ | M2 | 166,035 | H | 61,334 | 533.8 | 42,860 | 34,298 | 56% |
|  | 3G66_A_ES-Fc |  |  | K | 77,445 | 498.5 | 71,133 | 55,838 | 72% |
| 9 | Unimmunized | N | 222,491 | H | 42,391 | 523.7 | 28,795 | 22,365 | 53% |
|  |  |  |  | K | 150,144 | 501.3 | 139,580 | 111,437 | 74% |
| Listed items include the library index, antigen name, mouse index, total number of raw reads, antibody chain type, number of antibody chains, average read length, number of full-length antibody chain sequences after pipeline processing, number of usable sequences after filtering with a cutoff of 250bp for V-gene alignment, and the percentage of usable sequences. | | | | | | | | | |
| IgG and IgM frequency analysis | | | | | | | | | |
| Library index | Antigen | N_assign_ | IgG1 | IgG2b | IgG2c | IgG3 | IgM | IgG (%) | IgM (%) |
| 1 | 1GUT_A_ES-FR | 42,606 | 5 | 4 | 12 | 6 | 42,579 | 0.06 | 99.94 |
| 2 | 1KIG_L_ES-2-FR | 148,148 | 1,199 | 125 | 356 | 267 | 146,201 | 1.31 | 98.69 |
| 3 | gp140.664.R1 | 103,807 | 21 | 32 | 42 | 23 | 103,689 | 0.11 | 99.89 |
| 4 | gp140.664.R1/  1GUT_A_ES-FR | 55,827 | 33 | 23 | 30 | 26 | 55,715 | 0.20 | 99.80 |
| 5 | 3O0P_A_ES-Fc | 173,927 | 25 | 32 | 22 | 25 | 173,823 | 0.06 | 99.94 |
| 6 | 3G66_A_ES-Fc | 44,552 | 1 | 2 | 9 | 6 | 44,534 | 0.04 | 99.96 |
| 7 | gp140.681.R1-1NOG | 151,481 | 159 | 113 | 69 | 109 | 151,031 | 0.30 | 99.70 |
| 8 | gp140.681.R1-1NOG/  3G66_A_ES-Fc | 47,620 | 9 | 8 | 9 | 13 | 47,581 | 0.08 | 99.92 |
| 9 | Unimmunized | 33,815 | 6 | 10 | 9 | 10 | 33,780 | 0.10 | 99.90 |
| Listed items include the library index, antigen name, number of heavy chains that can be reliably assigned to a germline V gene (obtained from step 2 of the pipeline processing), number of IgG1, IgG2b, IgG2c, IgG3, and IgM sequences, as well as the percentages of IgG and IgM in the total repertoire. | | | | | | | | | |
